# Emulsifiers in ultra-processed foods in the UK food supply

**DOI:** 10.1017/S1368980023002021

**Published:** 2023-11

**Authors:** Alicia Sandall, Leanne Smith, Erika Svensen, Kevin Whelan

**Affiliations:** King’s College London, Department of Nutritional Sciences, Franklin Wilkins Building, London, SE1 9NH, UK

**Keywords:** Ultra-processed food, Food additive, Emulsifier, Food database

## Abstract

**Objective:**

Ultra-processed foods (UPF), including those containing food additive emulsifiers, have received research attention due to evidence implicating them in the pathogenesis of certain diseases. The aims of this research were to develop a large-scale, brand-level database of UPF in the UK food supply and to characterise the occurrence and co-occurrence of food additive emulsifiers.

**Design:**

A database was compiled sampling all products from the food categories contributing to energy intake from UPF in the UK from the National Diet and Nutrition Survey (2008–2014). Every food in these categories were identified from online supermarket provision from the ‘big four’ supermarkets that dominate the market share in the UK, comprising Tesco, Sainsbury’s, Asda and Morrisons.

**Setting:**

Major supermarkets in the UK.

**Results:**

A total of 32 719 food products in the UK supermarket food supply were returned in searches. Of these, 12 844 products were eligible and manually reviewed for the presence of emulsifiers. Emulsifiers were present in 6642 (51·7 %) food products. Emulsifiers were contained in 95·0 % of ‘Pastries, buns and cakes’, 81·9 % of ‘Milk-based drinks’, 81·0 % of ‘Industrial desserts’ and 77·5 % of ‘Confectionary’. Fifty-one per cent of all emulsifier-containing foods contained multiple emulsifiers. Across emulsifier-containing foods, there were a median of two emulsifiers (IQR 2) per product. The five most common emulsifiers were lecithin (23·4 % of all products), mono- and diglycerides of fatty acids (14·5 %), diphosphates (11·6 %), and xanthan gum and pectin (8·0 %).

**Conclusions:**

Findings from this study are the first to demonstrate the widespread occurrence and co-occurrence of emulsifiers in UPF in the UK food supply.

Food additives are substances that are not normally consumed as food itself but added to food intentionally for a technological purpose^(1)^. Since the 1950s, ready-to-eat, convenience packaged products containing food additives have become more prominent in the food supply^(2)^.

Ultra-processed foods (UPF) are a group of foods defined based on the extent and purpose of processing as part of the NOVA classification system adopted by the FAO of the UN^(2)^. The NOVA classification defines UPF as those whose constituent ingredients are of exclusive industrial use, usually created by a sequence of processing techniques only available in industry^(2)^.

Dietary surveys have demonstrated that UPF contribute to 16 %–58 % of total energy intake^(3)^. In the UK, UPF contribute to over half the average UK daily adult energy intake^(4)^. In Europe, household purchasing of UPF is highest in the UK, contributing 50·4 % of household foods^(5)^. This significant share of UPF to supply and intake demonstrates how ubiquitous this food class is in the UK diet, a pattern observed in other high-income countries, such as the USA^(6)^, Canada^(7)^ and Australia^(8)^. UPF represent 83 % of packaged supermarket foods in New Zealand^(9)^, and 67 % in France^(10)^.

UPF have received extensive recent research attention due to epidemiological evidence of associations with disease. For example, prospective cohort studies have associated increased UPF consumption with overweight and obesity^(11)^, hypertension^(12)^, cancer^(13)^, Crohn’s disease^(14)^ and all-cause mortality^(15)^. Some UPF contain high amounts of added fat, sugar and/or salt^(16,17)^ in addition to food additives, all of which are being mechanistically implicated in the aetiology of some of these disorders^(18)^.

Emulsifiers are a family of food additives present in a wide range of UPF, including industrial sliced bread, bakery foods, ready-made sauces, chocolate, confectionary and processed dairy foods^(19)^. Emulsifiers form or stabilise a uniform emulsion of two or more phases in a food^(19)^. For example, they can be used to maintain a uniform consistency in fat-containing foods that would otherwise form an unappetising separation of oil and water. However, recent evidence has implicated food additive emulsifiers in disease pathogenesis of metabolic syndrome^(18)^ and inflammatory bowel disease (IBD)^(20)^. In murine models, emulsifiers modify the luminal and mucosal microbiome and increase intestinal and chronic low-grade systemic inflammation^(21–23)^.

Measuring the presence of emulsifiers in the food supply is important as population exposure patterns are crucial to understanding potential deleterious health effects of emulsifiers. Depending on the body responsible for legislating food additives, there are between 63 and 261 emulsifiers added to foods throughout the world, with sixty-six in the UK as defined by both the Codex Alimentarius and the Joint FAO and WHO Expert Committee on Food Additives^(19)^. However, there is a paucity of research regarding the foods in which emulsifiers are contained, and so the occurrence of food additive emulsifiers in the UK food supply is unknown^(24)^, in part due to the absence of a database of emulsifier content of foods that lists all UPF and details all different types of emulsifiers. Such a database would enable analysis of food additive emulsifier occurrence in the food supply and enable measurement of the frequency of emulsifier exposure in dietary surveys and clinical trials.

The aims of this research were to develop a large-scale, brand-level database of UPF in the UK food supply and to characterise the occurrence and co-occurrence of food additive emulsifiers.

## Methods

This study involved the development of a database of the majority of UPF in the UK food supply, and then extraction of data on the presence and type of emulsifier from manual review of all ingredients labels.

### Eligibility of foods

Eligibility criteria for foods were developed *a priori* using the FAO definition of UPF and National Diet and Nutrition Survey descriptors^(2,25)^ (Table 1).

**Table 1:** Search terms and eligibility criteria for the fifteen top consumed ultra-processed food (UPF) categories in the UK. Adapted using the FAO descriptors of ultra-processed foods and National Diet and Nutrition Survey descriptors

Food category contributing to UPF intake in UK		Search term(s)	Included	Excluded
1	Industrialised packaged breads	Bread	All industrialised packaged breads including sliced bread, soda bread, bagels, pitta, baguettes, bread rolls, chapatis, naan, garlic bread, English muffins, wheat tortillas/wraps, brioche and part-baked packaged bread. Gluten-free versions of these breads were included.	Pizza, croutons, waffles, bread sauce mix, bread sticks, bread flour, bread and butter pudding, breadcrumbs, crisp bread crackers, bruschetta, poppadums, scones, hot cross buns, tea cakes, fruit loaf, and malt loaf.
2	Packaged pre-prepared meals	Ready meal	All fresh or frozen packaged pre-prepared meals. Savoury meat pies were included (but reconstituted meat products on their own belong in category 4).	Packaged pre-prepared dishes that are side dishes (such as creamed carrots). Infant weaning ready meals. Nutritionally complete meal replacement drinks. Industrial pizza (category 14).
3	Breakfast cereals	Breakfast cereal	All breakfast cereals including porridge pots, breakfast slices (e.g. Kellogg’s® pop tarts), breakfast cereal bars (e.g. Kellogg’s Special K® bars and Alpen® breakfast bars) and breakfast biscuits (e.g. Belvita® breakfast biscuits).	Breakfast drinks (e.g. Weetabix® on-the-go drinks). Infant breakfast cereal (e.g. Aptamil® breakfast cereal 7 months+). Plain oats.
4	Sausage and other reconstituted meat products	Meat	Chilled, cured, frozen or canned reconstituted meat. Examples include sausage meat, chicken Kievs, burgers, meat balls, nuggets, pate, black/white pudding, pepperoni, pastrami, corned beef, luncheon meat, meat paste, faggots and haggis.	Meats that have not been reconstituted (e.g. pre-prepared sliced chicken breast or minced meat) or meats that have been cured but not reconstituted (e.g. gammon or bacon). Meat alternatives. Fish and reconstituted fish. Reconstituted meats for pet foods. Reconstituted meat as part of a ready meal (e.g. savoury meat pie, category 2).
5	Confectionary	Sweets Chocolate	Boiled sweets, gums, pastilles, fudge, chews, mints, rock, liquorice, toffees, nougat, chocolate bars, filled bars, chocolate wafer bars, assortments, marshmallow-based products (e.g. Tunnock’s® teacakes), Florentines, chewing gum, diabetic chocolate and chocolate-coated nuts/raisins.	Cereal bars or rice/corn cakes with chocolate (category 6).
6	Biscuits	Biscuit	All sweet and savoury biscuits, chocolate biscuits, Jaffa cakes, cereal bars, flapjacks, ice cream cornet/wafers, cream crackers, bread sticks, oat/rice cakes and macaroons. Gluten-free versions of these biscuits were included.	Cereal bars that are 100 % dried fruit/nut (e.g. Nakd® bars). Infant weaning biscuits.
7	Pastries, buns and cakes	Pastry Cake	All sweet pastry, pies and buns, pies. This includes Danish pastries, currant bun, doughnuts, American muffins, Eccles cakes, Bakewell tarts, jam tarts, scones (sweet and savoury), sponge cakes, fruit cakes, eclairs, fruit loaf, malt loaf, gateaux, pastry, mince pies, sponge fingers, scotch pancakes, croissants, custard tart, lemon meringue pie, egg custard, caramel shortcake and strudel. Ready-to-roll chilled/frozen pastry.	Chilled set puddings where the major component is not pastry/cake (e.g. jelly-set trifle, Tiramisu, cheesecake and banoffee pie).
8	Industrial chips (i.e. oven chips and French fries)	Chips	French fries, croquettes, potato smileys, potato waffles and hash brown. Products may be fresh/frozen and oven/microwave preparations.	Mashed potato powder (e.g. Smash®).
9	Soft drinks, fruit drinks and fruit juices	Soft drink Fruit juice	All fruit juice, squashes, cordial.	Blended fruit-based drinks that contain milk (category 10). Alcohol-free or low alcohol wines/beers/ciders.
10	Milk-based drinks	Milkshake Smoothie	Milk-based drinks based on animal milks. Milkshakes or smoothies marketed as high protein drinks.	Plain milk (whether pasteurised, unpasteurised or ultra-heat treated). Infant formula (powder or ready-to-drink). Milk powders (e.g. milk/milkshake powders). Milkshakes or smoothies made from dairy-free, alternative milks. Meal replacement powders.
11	Margarine and other spreads	Margarine Spread	Margarine, cooking spread and butter alternatives (e.g. vegan spread and dairy-free spread).	Butter, spreadable cheese, other cooking fats and oils (lard and dripping), fish- and meat-based spread (e.g. pate, dips and sandwich filling).
12	Sauces, dressings and gravies	Sauce Dressing Gravy Condiment Vinaigrette	White sauces, cook-in-sauces (pesto, stir through sauces and pasta sauces), sauce mixes, tomato ketchup, Bovril/Marmite, chutney (including Branston pickles and Piccalilli), gravy, mayonnaise, salad cream and dressings (e.g. thousand island dressing, sweet chilli, Worcester, brown, HP, mint and sriracha) and stock (dried, concentrate and liquid)	Pies, packaged ready meals, dried herbs and spices (including spice mixes and rubs), tomato puree, stuffing (dried or fresh), yeast, pickles (pickled vegetables such as onions, gherkins, kimchi and sauerkraut), dessert syrups and spreads (chocolate, strawberry and toffee). Foods in which sauce is not the main component of the product (e.g. beans in sauce, tinned fish in sauce and pasta ‘n’ sauce).
13	Packaged salty snacks	Crisps Pretzels Popcorn	Vegetable and legume-based crisps (such as potato, maize, lentil and chickpea), popcorn (not sweet), twiglets, tortilla chips/snacks, pork scratchings, savoury flavoured rice snacks and Bombay mix.	Sweet popcorn, tortillas wraps, naan bread, nuts and seeds, yogurt and chocolate-coated rice snacks, biscuits.
14	Industrial pizza	Pizza	Frozen pizza, ready-made pizza, pizza bases and pizza baguettes/rolls.	Pizza sauces, pizza dough, dough balls and pizza-flavoured crisps/nachos.
15	Industrial desserts	Dessert Ice cream	Dairy-based desserts, ice cream, mousses, chilled set puddings (such as jelly-set trifle, tiramisu, cheesecake and banoffee pie), custard, flavoured yogurt, squirty cream, dessert sauces (maple syrup, strawberry, chocolate and caramel) and ice cream roll/ice cream filled buns.	Confectionary (chocolate and pastilles), fresh/frozen fruit, cream, crème fraiche, evaporated milk, biscuits, cakes, sweet pies and pastries, plain yogurt, savoury puddings (Yorkshire/or meat), wafers and ice cream cones.

Food categories are the top fifteen that contribute to UPF intake in the UK based on National Diet and Nutrition Survey data^(4)^. The search terms and eligibility are adapted from FAO descriptors of UPF^(2)^ and the National Diet and Nutrition Survey categories^(25)^.

A database was compiled of all products in the food categories that contributed to UPF intake from the National Diet and Nutrition Survey (2008–2014)^(4)^. The top fifteen food categories contributing to energy intake from UPF in the UK were as follows: industrialised packaged breads (11·01 % of total energy intake); packaged pre-prepared meals (7·77 %); breakfast cereals (4·36 %); sausage and other reconstituted meat products (3·84 %); confectionery (3·55 %); biscuits (3·46 %); pastries, buns and cakes (3·26 %); industrial chips (French fries) (2·79 %); soft and fruit drinks (2·49 %); milk-based drinks (2·23 %); margarine and other spreads (2·19 %); sauces, dressings and gravies (2·11 %); packaged salty snacks (2·02 %); industrial pizza (1·84 %); and industrial desserts (0·87 %)^(4)^. Thus, the database consisted of all foods in these categories that are known to contribute 53·79 % of mean adult energy intake in the UK^(4)^.

### Search strategy

Every food item in these top fifteen categories contributing to UPF intake were identified from an online search of supermarkets. The supermarket websites of the ‘big four’ supermarkets that dominate the market share in the UK were reviewed, comprising Tesco (26·9 % market share), Sainsbury’s (14·9 %), Asda (13·9 %) and Morrisons (10·1 %) (data in the 12 weeks ending 14/06/2020 when the supermarket sample was selected)^(26)^.

A search term for each food category was devised (Table 1). As online supermarkets organise products into hierarchical ontologies, the search terms were chosen at the highest level in order to maximally widen the search (e.g. ‘bread’ would capture white loaf, tortilla, wraps, ciabatta, etc). Where food categories had broad descriptors (e.g. confectionary), numerous search terms were selected (e.g. sweets and chocolate) to maximise identification of individual food items^(25)^.

The search terms were used in the websites of the four supermarkets to retrieve all food products in the UK food supply in that category. The supermarket search was conducted in Google Chrome used in ‘incognito mode’ to minimise the influence of previous browsing history on retrieval of food brands from the search results^(27)^. Searches were conducted over a 6-month period (June 2020–November 2020) and was performed by a team of three research dietitians.

### Data extraction for emulsifier content

All retrieved food products from each supermarket search were transferred into the database ready for de-duplication, eligibility screening and data extraction.

Identical food products were identified, and duplicates discounted from the analysis (e.g. four identical brands of tomato ketchup in identical portion size, one from each of the supermarkets). Differing portion sizes of the same food products were not excluded on the basis of duplication (given the individual products remained representative of different products available in the food supply). After discounting duplicates, each food product was assessed for eligibility based on inclusion criteria (Table 1).

In the final count of eligible food products, the ingredients labels were extracted from supermarket database and manually reviewed for the presence of food additive emulsifiers, and the details extracted. The list of sixty-six food additives classified as emulsifiers was based on both JECFA and Codex Alimentarius classifications and have been published elsewhere^(19)^, a list of which are provided for reference in Supplemental Table 1, including the International Numbering System code (‘E number’) and full name of the food additive emulsifier.

Thus, the final database contained the following extracted data: food item brand name; food category (e.g. industrialised breads); search term under which it was identified, eligibility (yes/no); ingredients list for eligible food items; and emulsifier details (presence/absence, which emulsifiers, total number of emulsifiers).

De-duplication, eligibility screening and data extraction were performed by the same team of three research dietitians. In order to ensure consistency of data extraction, the lead dietitian (PhD in emulsifiers and the gut) trained the team on the process and regularly reviewed data extraction in the early stages until the other team members developed independence. Following full data extraction, database checking was performed on twenty randomly selected food items from each of the fifteen food categories (300 food items in total). Of the 300 random food items checked, there were no errors or disputes in de-duplication, eligibility screening or data extraction for emulsifiers.

### Statistical analysis

Frequency of emulsifiers are presented as median and interquartile range (IQR) due to non-normal distribution, and the number of emulsifiers in food products across UPF categories were compared using a Kruskal–Wallis test. Categorical data are presented as *n* (%) and were compared across UPF groups using Chi-square tests. Emulsifier co-occurrence was investigated using Spearman’s rank correlation and presented as a heat map. For all statistical tests, a *P*-value of <0·05 was considered statistically significant.

## Results

### Supermarket food product sample

**Fig. 1:**
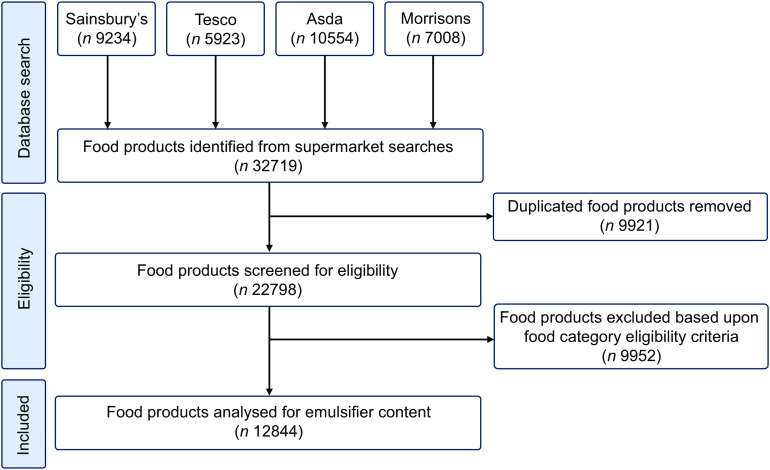
Flow diagram of food product inclusion

A total of 32 719 food products were returned in searches. Of these, 9921 food products were excluded based on duplication and a further 9954 excluded based on ineligibility (Fig. 1). Therefore, the ingredients of 12 844 food products (both foods and beverages) in the UK supermarket food supply were manually reviewed for the presence of food additive emulsifiers.

### Occurrence of emulsifiers

Overall, food additive emulsifiers were present in 6642 (51·7 %) of the 12 844 foods reviewed (Table 2).

**Table 2:** Occurrence of food additive emulsifiers by ultra-processed food (UPF) category in the UK food supply

Ultra-processed food category	Products assessed, *n*	Products with at least one emulsifier, *n*	%	Products with multiple emulsifiers, n (% of products assessed, % of products with emulsifiers)	%	Emulsifiers per emulsifier-containing products, median (IQR)	IQR	Maximum emulsifiers in a product, *n*	Top fiv most common emulsifiers, E-number *n*	%
Total UPF	12 844	6642	51·7	3397	26·4, 51·1	2	2	11	E322 3010 E471 1862 E450 1496 E415 1058 E440 1025	23·4 14·5 11·6 8·2 8·0
Industrialised packaged breads	640	399	62·3	219	34·2, 54·9	2	1	4	E472e 224 E471 187 E481 88 E464 62 E415 47	35·0 29·2 13·8 9·7 7·3
Packaged pre-prepared meals	1410	364	25·8	101	7·2, 27·7	1	1	6	E440 102 E450 82 E471 57 E415 45 E412 41	7·2 5·8 4·0 3·2 2·9
Breakfast cereals	505	115	22·8	30	5·9, 26·1	1	1	5	E322 96 E440 17 E450, 15 E472e 11 E471 8	19·0 3·4 3·0 2·2 1·6
Sausages and other reconstituted meat products	1015	474	46·7	172	16·9, 36·3	1	1	6	E450 356 E452 79 E407 50 E412 34 E404 31	35·1 7·8 4·9 3·3 3·1
Confectionary	2083	1615	77·5	558	26·8, 34·6	1	1	7	E322 1314 E476 276 E442 252 E471 204 E414 132	63·1 13·3 12·1 9·8 6·3
Biscuits	874	557	63·7	286	32·7, 51·3	2	1	6	E322 456 E450 160 E476 86 E440 62 E471 59	52·2 18·3 9·8 7·1 6·8
Pastries, buns and cakes	823	782	95·0	690	83·8, 88·2	4	3	10	E471 606 E450 568 E322 384 E440 265 E415 240	73·6 69·0 46·7 32·2 29·2
Industrial chips	142	41	28·9	10	7·0, 24·4	1	0	3	E415 23 E450 15 E464 8 E461 3 E471 3	16·2 10·6 5·6 2·1 2·1
Soft drinks, fruit drinks and fruit juices	644	104	16·1	30	4·7, 28·8	1	1	5	E440 40 E445 25 E414 23 E415 19 E466 19	6·2 3·9 3·6 3·0 3·0
Milk-based drinks	160	131	81·9	92	57·5, 70·2	2	2	5	E407 101 E466 64 E460 56 E412 18 E415 14	63·1 40·0 35·0 11·3 8·8
Margarine and other spreads	101	61	60·4	26	25·7, 42·6	1	1	4	E322 42 E471 42 E404 11 E476 5 *No others*	41·6 41·6 10·9 5·0
Sauces, dressings and gravies	1833	543	29·6	119	6·5, 21·9	1	0	4	E415 380 E412 93 E322 99 = E405 25 = E440 25	20·7 5·1 5·4 1·4 1·4
Packaged salty snacks	767	89	11·6	10	1·3, 11·2	1	0	3	E471 40 E322 30 E414 9 E415 7 E472E 3	5·2 3·9 1·2 0·9 0·4
Industrial pizza	332	140	42·2	66	19·9, 47·1	1	1	5	E450 53 E452 52 E415 25 E464 22 E412 18	16·0 15·7 7·5 6·6 5·4
Industrial desserts	1515	1227	81·0	988	65·2, 80·5	1	2	11	E471 596 E412 549 E322 542 E410 534 E440 405	39·3 36·2 35·8 35·2 26·7
*P*-value		<0·001		<0·001		<0·001				

Ultra-processed foods are defined as detailed by Rauber *et al.* (2018) according to the FAO NOVA definitions^(4)^. Emulsifiers are identified by E-number (the Codex Alimentarius International Numbering System number, prefixed by the letter ‘E’)^(49)^. Number of emulsifiers per food product (median, IQR) was assessed for significance between UPF categories using a Kruskal–Wallis test. Proportions of food products containing emulsifiers between UPF categories was assessed using Chi-square tests for independence.

The five most common emulsifiers across all products were lecithin (E322) (23·4 % of all UPF), mono- and diglycerides of fatty acids (E471) (14·5 %), diphosphates (E450) (11·6 %), xanthan gum (8·2 %) and pectin (E440) (8·0 %) (Table 2).

From the sixty-six food additive emulsifiers approved for use in the EU, fifty-one were identified in the products reviewed. Due to foods containing multiple emulsifiers (Table 2), there were 14 300 occurrences of emulsifiers across 6642 emulsifier-containing foods. Patterns of occurrence of individual emulsifiers are shown in Table 3.

**Table 3:** Occurrence of individual emulsifiers across ultra-processed food categories in the UK food supply

Emulsifier E-number	Frequency of emulsifier occurrence across ultra-processed food categories, n																
	1 Breads	2 Prepared meals	3 Breakfast cereal	4 Sausage, meat products	5 Confectionary	6 Biscuits	7 Pastries, buns, cakes	8 Chips	9 Soft drinks, fruit drinks, juices	10 Milk drinks	11 Margarine, spreads	12 Sauces, dressings, gravy	13 Salty snacks	14 Pizza	15 Desserts	Total occurrence, *n*	%
E322(i-iii)	18	9	96	18	1314	456	384	0	0	0	42	99	30	2	542	3010	21·0
E339(i-iii)	1	28	0	5	3	10	18	0	1	7	0	1	1	2	32	109	0·8
E400	0	0	0	0	0	0	0	0	0	0	0	0	0	0	1	1	0·0
E401	1	2	0	12	7	3	89	0	0	0	0	2	0	0	108	224	1·6
E402	2	0	0	0	0	0	0	0	0	0	0	0	0	0	3	5	0·0
E403	0	0	0	0	0	0	0	0	0	0	0	0	0	0	0	0	0·0
E404	0	0	0	31	0	0	21	0	0	0	11	0	0	0	9	72	0·5
E405	0	0	0	0	0	0	0	0	0	0	0	25	0	0	0	25	0·2
E406	2	11	0	2	6	1	35	0	0	0	0	3	0	4	44	108	0·8
E407	0	27	2	50	26	3	29	1	1	101	0	4	0	12	309	565	4·0
E407a	0	0	0	9	0	0	0	0	0	0	0	0	0	0	3	12	0·1
E410	0	8	0	7	10	13	16	0	1	4	0	15	0	4	534	612	4·3
E412	13	41	3	34	2	9	30	2	15	18	0	93	0	18	549	827	5·8
E413	0	0	0	0	1	1	85	0	0	0	0	1	0	0	4	92	0·6
E414	0	12	3	1	132	15	62	0	23	3	0	1	9	7	27	295	2·1
E415	47	45	5	24	12	34	240	23	19	14	0	380	7	25	183	1058	7·4
E416	0	0	0	0	0	0	0	0	0	0	0	0	0	0	0	0	0·0
E423	0	0	0	0	0	0	0	0	0	0	0	0	0	0	0	0	0·0
E425	0	0	0	0	0	3	0	0	0	0	0	0	0	0	2	5	0·0
E426	0	0	0	0	0	0	0	0	0	0	0	0	0	0	0	0	0·0
E427	0	0	0	0	0	0	0	0	0	0	0	0	0	0	0	0	0·0
E431	0	0	0	0	0	0	0	0	0	0	0	0	0	0	0	0	0·0
E432	0	0	0	0	0	0	0	0	0	0	0	0	0	0	0	0	0·0
E433	0	1	0	3	0	0	2	0	0	0	0	0	0	0	2	8	0·1
E434	0	0	0	0	0	1	0	0	0	0	0	0	0	0	0	1	0·0
E435	0	0	0	0	1	0	34	0	0	0	0	0	0	0	1	36	0·3
E436	0	0	0	0	0	0	0	0	0	0	0	0	0	0	0	0	0·0
E440	0	102	17	13	92	62	265	0	40	4	0	25	0	0	405	1025	7·2
E442	0	0	0	0	252	28	12	0	0	0	0	0	1	0	53	346	2·4
E445	0	0	0	1	0	1	0	0	25	0	0	0	0	0	0	27	0·2
E450 (i-ix)	35	82	15	356	21	160	568	15	0	0	0	5	8	53	178	1496	10·5
E452 (i-vi)	1	26	0	79	3	2	2	0	3	0	0	8	0	52	26	202	1·4
E460 (i-ii)	2	5	0	3	4	5	0	0	0	56	0	0	0	2	0	77	0·5
E461	3	19	3	4	0	0	5	3	0	0	0	1	2	17	0	57	0·4
E462	0	0	0	0	0	0	1	0	0	0	0	0	0	0	0	1	0·0
E463	7	0	0	0	0	0	0	0	0	0	0	0	0	0	2	9	0·1
E464	62	18	0	4	0	1	21	8	0	0	0	1	0	22	1	138	1·0
E465	0	0	0	0	0	0	0	0	0	0	0	0	0	0	0	0	0·0
E466	20	5	0	1	16	1	24	0	19	64	0	2	0	0	27	179	1·3
E467	0	0	0	1	0	0	0	0	0	0	0	0	0	0	0	1	0·0
E468	0	0	0	0	0	0	1	0	0	0	0	0	0	0	0	1	0·0
E469	0	0	0	0	0	1	0	0	0	0	0	0	0	0	0	1	0·0
E470 (i-iii)	0	0	0	4	16	0	10	0	0	0	0	0	0	0	0	30	0·2
E471	187	57	8	25	204	59	606	3	0	12	42	6	40	17	596	1862	13·0
E472a	0	0	0	0	0	3	4	0	0	0	0	0	0	0	3	10	0·1
E472b	0	0	1	0	3	2	35	0	0	0	0	0	0	1	95	137	1·0
E472c	0	0	0	0	0	1	8	0	0	1	0	0	0	0	6	16	0·1
E472d	0	1	0	0	0	0	33	0	0	0	0	1	0	0	4	39	0·3
E472e	224	15	11	6	1	25	3	0	0	0	0	0	3	4	7	299	2·1
E472f	0	0	0	0	0	0	9	0	0	0	0	0	0	2	0	11	0·1
E473	0	8	0	0	17	8	17	0	0	0	0	0	0	0	1	51	0·4
E474	0	0	0	0	0	0	0	0	0	0	0	0	0	0	0	0	0·0
E475	2	6	7	0	4	14	231	0	0	0	0	0	0	3	66	333	2·3
E476	2	0	0	0	276	86	32	0	0	0	5	0	1	0	82	484	3·4
E477	0	1	0	0	0	0	37	0	0	0	0	0	0	0	32	70	0·5
E478	0	0	0	0	0	0	0	0	0	0	0	0	0	0	0	0	0·0
E479	2	0	0	0	0	0	0	0	0	0	0	0	0	0	0	2	0·0
E481 (i-ii)	88	1	0	0	0	7	133	0	0	0	0	0	0	0	7	236	1·7
E482 (i-ii)	1	0	0	0	0	0	0	0	1	0	0	0	0	0	0	2	0·0
E491	0	0	0	0	0	0	22	0	0	0	0	0	1	2	0	25	0·2
E492	0	0	1	0	29	8	26	0	0	0	0	0	0	0	2	66	0·5
E493	0	0	0	0	0	0	0	0	0	0	0	0	0	0	0	0	0·0
E494	0	0	0	0	0	0	0	0	0	0	0	0	0	0	0	0	0·0
E495	0	0	0	0	0	0	0	0	0	0	0	0	0	0	0	0	0·0
E541	0	0	0	0	0	0	0	0	0	0	0	0	0	0	0	0	0·0
E999(i-ii)	0	0	0	0	0	0	0	0	2	0	0	0	0	0	0	2	0·0
																Total 14 300	

Emulsifiers are identified by their E-number (the Codex Alimentarius International Numbering System number, prefixed by the letter ‘E’)^(49)^ and the number of food products containing that emulsifier are reported for each of the top fifteen UPF categories contributing to energy intake in the UK^(4)^.

Of the emulsifiers with most research on their impact on the gastrointestinal tract, carrageenan was present in 565 foods (4·4 %), E466 in 179 foods (1·4 %) and polysorbate-80 in eight foods (0·06 %).

### Occurrence of emulsifiers by food category

Overall, 51·7 % of the foods contained food additive emulsifiers; however, there was wide variation and statistically significant differences between UPF categories (*P* < 0·001). For example, emulsifiers were contained in 95·0 % of ‘Pastries, buns and cakes’, 81·9 % of ‘Milk-based drinks’, 81·0 % of ‘Industrial desserts’ and 77·5 % of ‘Confectionary’. In contrast, they were contained in only 11·6 % of ‘Packaged salty snacks’ (Table 2).

The type of emulsifier was also highly variable across food categories, for example, carrageenan (E407) was the most common emulsifier in ‘Milk-based drinks’ (63·1 % of products) and was only present in 4·9 % of ‘Sausage and other reconstituted meat products’ (Table 2). Carboxymethylcellulose (E466) was present in 40·0 % of ‘Milk-based drinks’ and only 3·0 % of ‘Soft drinks, fruit drinks and fruit juices’ (Table 2). Polysorbate-80 was only identified in eight foods (Table 3) from varying food categories.

### Frequency of emulsifiers in each food

**Fig. 2:**
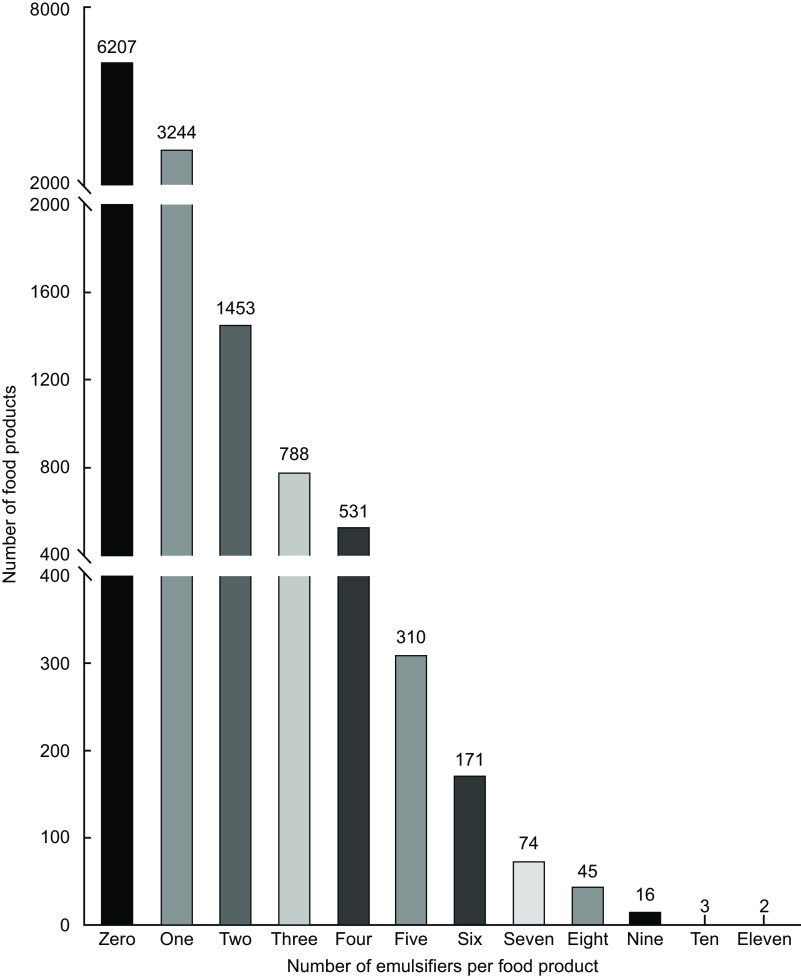
Number of individual emulsifiers per food product

Across all 6642 foods that contained an emulsifier, there were a median of two emulsifiers (IQR 2) per product (Table 2). Fifty-one per cent of emulsifier-containing foods contained multiple emulsifiers, but this pattern of co-occurrence varied between food categories (*P* < 0·001) (Table 2). For example, emulsifier-containing ‘Pastries, buns and cakes’ had a median of four emulsifiers (IQR 3) per product, and ‘industrialised packaged breads’, ‘biscuits’ and ‘milk-based drinks’ had a median of two emulsifiers per product. Of all emulsifier-containing foods, 11·3 % contained at least two emulsifiers, 6·1 % three, 4·1 % four and 4·8 % five or more emulsifiers (Fig. 2).

### Co-occurrence of emulsifiers in foods

**Fig. 3:**
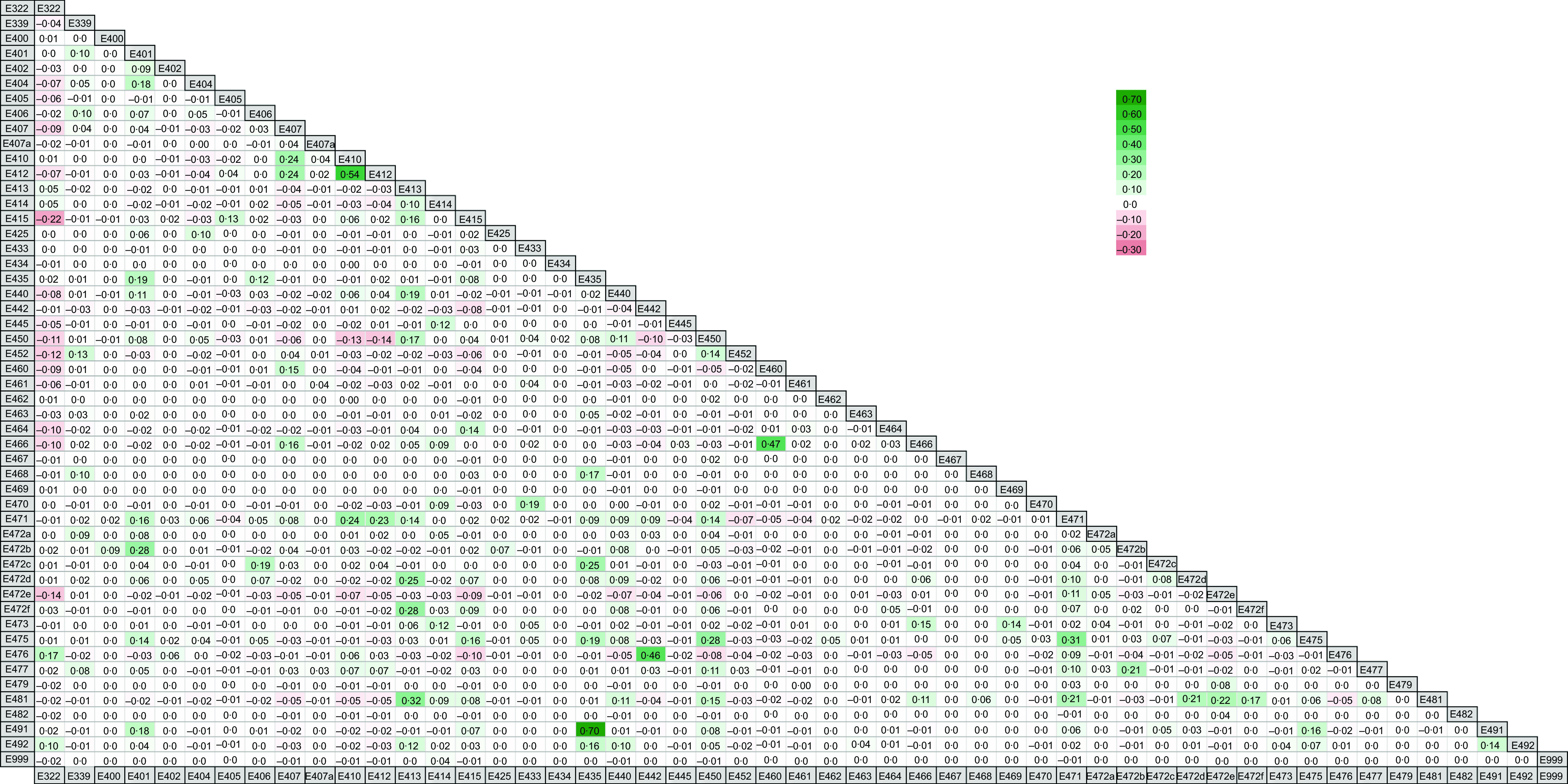
Heat map displaying co-occurrence of individual food additive emulsifiers in the UK food supply. Values are Spearman’s rho correlation coefficients, and cell shading indicates the magnitude of correlation co-occurrence between emulsifiers. The degree of green shading indicates positive correlations, and red shading denotes negative correlations

There were a number of statistically significant positive correlations (Spearman’s rank) between emulsifier co-occurrence in foods indicating common use of the two emulsifiers together in a food product (Fig. 3). There were strong positive correlations between the presence of polysorbate-60 (E435) and sorbitan monostearate (E491) (rho = 0·70, *P* < 0·001) and between carob bean gum (E410) and guar gum (E412) (rho = 0·54, *P* < 0·001). There were moderate positive correlations between the presence of ammonium salts of phosphatidic acid (E442) and polyglycerol esters of interesterified ricinoleic acid (E476) (rho = 0·46, *P* < 0·001); sodium lactylates (E481) and tragacanth gum (E413) (rho = 0·32, *P* < 0·001); microcrystalline cellulose and powdered cellulose (E460) and carboxymethyl cellulose (E466) (rho = 0·47, *P* < 0·001); and E471 and E475 (rho = 0·31, *P* < 0·001).

There were a small number of statistically significant negative correlations between emulsifier co-occurrence, although in general these were weakly correlated (Fig. 3), for example, lecithin (E322) and xanthan gum (E415) (rho = −0·22, *P* < 0·001), indicating that the presence of one was associated with less use of the other.

## Discussion

This paper demonstrates the ubiquity of food additive emulsifiers across foods contributing to UPF intake in the UK food supply. Over half of foods in these UPF categories contained emulsifiers, being most commonly found in industrial desserts, milk-based drinks and confectionary, pastries and cakes. The most common emulsifiers were lecithin, mono- and diglycerides of fatty acids, diphosphates, xanthan gum and pectin; however, variations in emulsifier type occurred across food categories. Half of all emulsifier-containing foods contained more than one emulsifier, with two foods containing eleven emulsifiers.

Comparing the findings of the present study with previous literature is challenging as minimal data are available on the occurrence of emulsifiers in foods at the brand level. Studies attempt to measure either emulsifier intakes in population groups or emulsifier content in the food supply (as in the current study).

In terms of measuring emulsifier intakes, Shah *et al.,* (2017) estimated population exposure to seven common emulsifiers in the USA (carboxymethycellulose (E466), polysorbate-80 (E433), lecithin (E322), mono- and diglycerides of fatty acids (E471), sodium lactylates (E481), sucrose esters of fatty acids (E473) and polyglycerol esters of interesterified ricinoleic acid (E476)^(28)^. They combined national dietary survey data, including the 2003–2010 National Health and Nutrition Examination Survey (NHANES) (2-d dietary intake) and assumed all food categories contained the US Maximum Permitted Levels of each emulsifier^(28)^. Lecithin (55 mg/kg bw/d) and mono- and diglycerides of fatty acids (80 mg/kg bw/d) had the highest mean exposure, whereas exposure to carboxymethycellulose (27 mg/kg bw/d) and polysorbate-80 (8 mg/kg bw/d) was relatively low. Whilst a very different methodological approach was used to estimate emulsifier exposure by Shah *et al.,* (2017) (individual dietary intake data) compared to the present study (occurrence in the food supply), it mirrors the findings reported here that lecithin and mono- and diglycerides of fatty acids are more common than polysorbate-80 or carboxymethycellulose.

Other studies have measured emulsifiers in the food supply, similar to the current study. The United States Department of Agriculture Global Branded Food Products Database (241 688 food products) has been used to investigate patterns of food additive content across baked goods, reporting emulsifiers being present in 91 % of cookies, 94 % of crackers, 95 % of bread and rolls and 100 % of pastry and doughnuts^(29)^. Lecithin accounted for about 44–45 % of the total counts of emulsifiers listed in cookies and crackers, whereas gums such as xanthan, guar, and agar account for about 18 % of the listed emulsifiers in pastries. Whilst that study only measured food additives in one food group, it concurs with the findings presented here that emulsifiers are ubiquitous in baked goods. A database study in France using The Open Food Facts Database (126 566 food products) found that emulsifiers/thickeners were one of the most frequently used food additives, compared with antioxidants, dyes, preservatives and sweeteners^(30)^, with the most commonly present emulsifiers being lecithin (second most common food additive), xanthan gum (sixth), diphosphates (eighth), pectins (ninth) and mono- and diglycerides of fatty acids (tenth), which were identical to the five most common emulsifiers in the present study. In addition, carrageenan was common in the French food supply (thirteenth most common food additive, in 4·2 % of foods) and is similar to that in the current study (4·4 % of foods)^(30)^. Other studies have examined the presence of emulsifiers in food supply, although not at the level of individual emulsifiers as in the current study. A review of 24 229 foods in Australia reported emulsifiers to be the seventh most common ingredient being present in 15·6 % of all foods^(31)^, whilst a study of 9856 foods in Brazil reported emulsifiers to be the sixth most common food additive being present in 19·4 % of all foods^(32)^. Both of these values are lower than that reported here (51·7 %), although in the current study only foods contributing to UPF intake were selected and therefore our value is inevitably higher.

The emulsifiers implicated in gastrointestinal inflammation, carrageenan (4·4 % of foods), carboxymethycellulose (1·4 %) and polysorbate-80 (0·06 %) were not overall very common in the UK food supply. However, carrageenan and carboxymethylcelluose, which form a viscous solution in the aqueous phase creating a stable water-fat emulsion for a creamy mouthfeel^(33,34)^, were therefore contained in 63·1 % (carrageenan) and 40·0 % (carboxymethylcellulose) of all milk-based drinks. Thus, whilst these additives are not ubiquitous in the food supply, people consuming high levels of foods from these specific categories may be disproportionately exposed. A recent human study reported that carboxymethylcelluose reduced microbial diversity^(35)^; meanwhile, several prospective cohort studies report that higher intakes of UPF are associated with an increased risk of IBD^(14,36,37)^. Interestingly, different UPF categories, including soft drinks, refined sweetened foods, salty snacks and processed meat, were each independently associated with increased risk of IBD, suggesting that perhaps certain processing or food additives commonly found in these categories (such as the aforementioned emulsifiers) could theoretically be contributing to the development of IBD^(14)^.

Polysorbate-80 was rarely identified in food products in the present study. This could explain why the European Food Safety Authority (EFSA) estimates of mean intakes of polysorbates across Europe (0·6–16·9 mg/kg bw/d)^(38)^ are lower than that of carrageenan (22·0–88·9 mg/kg bw/d)^(39)^ and the modified celluloses (20–67 mg/kg bw/d)^(40)^.

This is the first study to demonstrate that UPF in the UK commonly contain multiple emulsifiers. Yet, whether emulsifiers have synergistic effects on gastrointestinal inflammation is not known, as previous murine and *ex vivo* studies tested individual emulsifiers in isolation^(21,22,41)^. Whilst half of all emulsifier-containing foods contained multiple emulsifiers, there were few strong patterns of co-occurrence. Co-occurrence of food additives is common across UPF. A third of foods in the United States Department of Agriculture Global Branded Food Products Database contain at least three additives^(42)^. The French-based Open Food Facts Database found that 11·6 % of food products contained at least two additives, 7·8 % three, 5·3 % four and 11·3 % five or more food additives^(30)^. This is similar to the present study, which found that 11·3 % of foods contained at least two emulsifiers, 6·1 % three, 4·1 % four and 4·8 % five or more emulsifiers. Of course, foods are often eaten together during a single meal, resulting in higher numbers, quantities and combinations of emulsifiers potentially being consumed within a single eating occasion. The NutriNet Santé cohort was used to identify six clusters of food additive consumption based on foods commonly consumed together (e.g. additives found in breakfast cereals, pastries and dairy desserts)^(43)^, emphasising that foods containing emulsifiers and other food additives are often eaten together and therefore exposure will depend not only on food supply but also on food preference, choice and meal pattern.

Fifteen food additives classified as emulsifiers were not present in the sample of foods. This could be due to a number of reasons. Firstly, it could be that these emulsifiers are used in foods categories not in the top fifteen most highly consumed UPF categories, although this would still result in low exposure. Secondly, it could be that food industry is reducing its use of certain emulsifiers. For example, soyabean hemicellulose (E426) which was not present in any food products in this study may be used less frequently, since soya allergy has become better understood and reported^(44)^. Another example is alginic acid (E400), a gel forming additive, that has been identified as a choking hazard in jelly confectionary owing to its semi-rigid consistency, and so its use in foods has been limited by the EU^(1)^.

This study was the first to explore the occurrence of all food additive emulsifiers in a large sample of foods in the UK food supply. The EFSA attempts to estimate population exposure to food additives in the EU, but current estimates use the rudimentary approach of assuming that all food categories that are permitted to contain emulsifiers will contain emulsifiers^(19)^. Whereas brand-level data with actual emulsifier occurrence would increase the external validity of population exposure estimates when combined with national dietary surveys, providing the surveys also record food intake at the brand level.

Emulsifier occurrence in this sample of 12 844 foods was considerable. This is significant because more than half of all foods in UK households are ultra-processed^5^, and these foods contribute to more than half of the UK energy intake^(4)^. If the deleterious effects of emulsifiers observed in animal models are confirmed in humans, and the present study has highlighted the ubiquity of emulsifiers in the food supply, then food formulations would require significant changes. This study is the first to confirm the widespread occurrence of food additive emulsifiers in the UK food supply and therefore the likely challenge of restricting dietary intakes of emulsifiers as part of ongoing therapeutic dietary interventions, for example, for metabolic syndrome^(23)^ or IBD^(20)^. Most concerningly, availability of UPF in middle-income countries are growing rapidly. Sales of UPF in South-East Asia and the East are expected to match those of high-income countries by 2035^(45)^.

### Strengths, limitations and future research

This is the first study to report the occurrence of emulsifiers in the UK food supply. It surveyed a large number of foods (12 844) contributing to UPF intake, larger than some previous studies in other countries^(29,32)^, although smaller than others that used pre-established databases^(30,31)^. Occurrence of total and individual emulsifiers was reported here, whereas some previous studies report only the occurrence of ‘total emulsifiers’^(29,31,32)^, which is important as there is escalating evidence of differential impacts of individual emulsifiers on gut health^(41,46)^.

A number of limitations of this study must be considered, and these mostly pertain to the UPF category sampling method.

Firstly, this study only sampled the top fifteen food categories, of the sixteen possible, that contribute to energy intake from UPF in the UK^(4)^. Therefore, it is likely other foods not sampled here represent further sources of emulsifiers in the UK food supply. The only UPF category not sampled, the sixteenth and final, was ‘Miscellaneous foods’^(4)^. This was because it would be challenging to search, identify and correctly categorise brand-level food items to a ‘miscellaneous’ category using supermarket websites. In addition, potentially eligible UPF in other categories were not searched. However, the top fifteen UPF categories sampled collectively contribute to 53·8 % of mean adult energy intake, and the remaining unaccounted miscellaneous category contribute only an additional 3·08 % to energy intake^(4)^.

Secondly, food items from only four supermarkets were analysed, and this does not represent the full range of food product availability. However, these ‘big four’ supermarkets collectively dominate the UK food supply, owning the total market share of 65·8 %^(26)^. Similarly, foods from restaurants or takeaways were not included in the analysis, and foods from these outlets may contain emulsifiers.

Thirdly, there is a risk the search terms did not retrieve all relevant foods, and so some foods in these fifteen UPF categories may have been missed. To minimise this, search terms were intentionally broad and inclusive in order to maximise the retrieval of results. Where it was perceived that a single search term would not be a catch-all term for a food group, multiple search terms for that food category were created *a priori* (Table 1).

Alternative data collection methodologies were considered that did not pose these three limitations in sampling approach. One option was to obtain the sample from market research data on the best-selling UPF products, as adopted to estimate phosphorus-based additives in the Australian food supply^(47)^ or using supermarket loyalty card data of food purchasing habits, as used to estimate protein intake in older adults in the UK^(48)^. The benefit of obtaining commercial data on UPF sales is that food products with a high population exposure are better captured in the sample frame. However, data on brand-level food sales was prohibitively costly, and this approach would not achieve the aim of this study, which related to measuring emulsifier occurrence in all foods contributing to UPF intake in the UK food supply.

Data were collected in the second half of 2020, just after the UK left the European Union (31 January 2020) but during the COVID-19 pandemic. Therefore, although the data collection period was entirely post-Brexit, its proximity to Brexit and to a global pandemic may mean that food supply measured at that time may have subsequently changed. Importantly, we surveyed four major supermarkets online (not in store) to minimise any supermarket-specific or localised food supply issues.

Future research may consider the nutrient composition, availability, and cost of emulsifier-free and emulsifier-containing foods to understand potential nutritional and economic impacts of consuming UPF-containing emulsifiers. In addition, the database can be used in future to identify other food additives in the UK food supply, and techniques that adopt algorithmic approaches to identifying these, rather than manual data extraction, could expedite this process.

## Conclusion

Findings from this study demonstrate the widespread occurrence of food additive emulsifiers in the UK supermarket food supply. Emulsifiers are present in 51·7 % of foods from UPF categories, the most common being lecithin, diphosphates and mono- and diglycerides of fatty acids. This is the first study to demonstrate that UPF in the UK commonly contain multiple emulsifiers, with 26·4 % containing two or more emulsifiers. The three emulsifiers of interest in gastrointestinal inflammation are present in relatively small numbers of UPF; however, 63·1 % and 40·0 % of milk-based drinks contained carrageenan and carboxymethylcelluose, respectively.

## Supplementary material

For supplementary material accompanying this paper visit https://doi.org/10.1017/S1368980023002021. click here to view supplementary material
