# Supplementary material for: Emulsifiers in ultra-processed foods in the UK food supply
Source: Public Health Nutr. 2023 Sep 21;26(11):2256–70. doi: 10.1017/S1368980023002021 (PMC10641632; doi:10.1017/S1368980023002021)
Supplement: Supplementary file 1 [file S1368980023002021sup.zip › S1368980023002021sup001.docx]

**Abstract**

**Objective:** Ultra-processed foods (UPF), including those containing food-additive emulsifiers, have received research attention due to evidence implicating them in the pathogenesis of certain diseases. The aims of this research were to develop a large-scale, brand-level database of UPFs in the UK food supply and to characterise the occurrence and co-occurrence of food-additive emulsifiers.

**Design:** A database was compiled sampling UPF groups contributing to total dietary energy intake in the UK from the National Diet and Nutrition Survey (2008-2014). Every food in these UPF groups were identified from online supermarket provision from the ‘‘big four’’ supermarkets that dominate the market share in the UK, comprising Tesco, Sainsbury’s, Asda and Morrisons.

**Results:** A total of 32,719 food products in the UK supermarket food supply were returned in searches. Of these, 12,844 UPF products were eligible and manually reviewed for the presence of emulsifiers. Emulsifiers were present in 6,642 (51.7%) food products. Emulsifiers were contained in 95.0% of ‘‘Pastries, buns and cakes’’; 81.9% of ‘‘Milk-based drinks”, 81.0% in ‘‘Industrial desserts’’ and in 77.5% of “Confectionary”. Fifty-one percent of all emulsifier-containing foods contained multiple emulsifiers. Across emulsifier-containing foods there were a median of 2 emulsifiers (IQR 2) per product. The five most common emulsifiers were lecithin (23.4% of all UPF), mono- and diglycerides of fatty acids (14.5%), diphosphates (11.6%), xanthan gum and pectin (8.0%).

**Conclusions:** Findings from this study are the first to demonstrate the wide-spread occurrence and co-occurrence of emulsifiers in UPF in the UK food supply.

## Introduction

Food additives are substances that are not normally consumed as food itself but added to food intentionally for a technological purpose^(1)^. Since the 1950s ready-to-eat, convenience packaged products containing food additives have become more prominent in the food supply^(2)^.

Ultra-processed foods (UPFs) are a group of foods defined based upon the extent and purpose of processing as part of the NOVA classification system adopted by the Food and Agriculture Organisation (FAO) of the UN^(2)^. The NOVA classification defines UPFs as those whose constituent ingredients are of exclusive industrial use, usually created by a sequence of processing techniques only available in industry^(2)^.

Dietary surveys have demonstrated that UPFs contribute to 16%-58% of total energy intake^(3)^. In the United Kingdom (UK), UPFs contribute to over half the average UK daily adult energy intake^(4)^. In Europe, household purchasing of UPFs is highest in the UK, contributing 50.4% of household foods^(5)^. This significant share of UPFs to supply and intake demonstrates how ubiquitous this food class is in the UK diet, a pattern observed in other high-income countries, such as the USA^(6)^, Canada^(7)^ and Australia^(8)^. UPFs represent 83% of packaged supermarket foods in New Zealand^(9)^, and 67% in France^(10)^.

UPFs have received extensive recent research attention due to epidemiological evidence of associations with disease. For example, prospective cohort studies have associated increased UPF consumption with overweight and obesity^(11)^, hypertension^(12)^, cancer^(13)^, Crohn’s disease^(14)^ and all-cause mortality^(15)^. Some UPFs contain high amounts of added fat, sugar and/or salt^(16,17)^ in addition to food-additives, all of which are being mechanistically implicated in the aetiology of some of these disorders^(18)^.

Emulsifiers are a family of food additives present in a wide range of UPFs, including industrial sliced bread, bakery foods, ready-made sauces, chocolate, confectionary and processed dairy foods^(19)^. Emulsifiers form or stabilise a uniform emulsion of two or more phases in a food^(19)^. For example, they can be used to maintain a uniform consistency in fat-containing foods that would otherwise form an unappetising separation of oil and water. However, recent evidence has implicated food-additive emulsifiers in disease pathogenesis of metabolic syndrome^(18)^ and IBD^(20)^. In murine models, emulsifiers modify the luminal and mucosal microbiome and increase intestinal and chronic low-grade systemic inflammation^(21, 22, 23)^.

Measuring the presence of emulsifiers in the food supply is important as population exposure patterns are crucial to understanding potential deleterious health effects of emulsifiers. Depending upon the body responsible for legislating food additives, there are between 63 and 261 emulsifiers added to foods throughout the world, with 66 in the UK as defined by both the Codex Alimentarius and the Joint FAO and World Health Organisation (WHO) Expert Committee on Food Additives^(19)^. However, there is a paucity of research regarding the foods in which emulsifiers are contained, and so the occurrence of food additive emulsifiers in the UK food supply is unknown^(24)^, in part due to the absence of a database of emulsifier content of foods that lists all UPFs and details all different types of emulsifiers. Such a database would enable analysis of food additive emulsifier occurrence in the food supply and enable measurement of the frequency of emulsifier exposure in dietary surveys and clinical trials.

The aims of this research were to develop a large-scale, brand-level database of UPFs in the UK food supply and to characterise the occurrence and co-occurrence of food-additive emulsifiers.

## Methods

This study involved the development of a database of the majority of UPFs in the UK food supply, and then extraction of data on the presence and type of emulsifier from manual review of all ingredients labels.

***Eligibility of UPF***

Eligibility criteria for UPFs were developed *a priori* using the FAO definition of UPF and National Diet and Nutrition Survey descriptors^(2,25)^ (**Table 1**).

A database was compiled using the UPF groups that contributed to energy intake from the National Diet and Nutrition Survey (2008-2014)^(4)^. The top 15 UPF groups contributing to total dietary energy intake in the UK were: industrialised packaged breads (11.01% of total energy intake); packaged pre-prepared meals (7.77%); breakfast cereals (4.36%); sausage and other reconstituted meat products (3.84%); confectionery (3.55%); biscuits (3.46%); pastries, buns, and cakes (3.26%); industrial chips (French fries) (2.79%); soft and fruit drinks (2.49%); milk-based drinks (2.23%); margarine and other spreads (2.19%); sauces, dressings and gravies (2.11%); packaged salty snacks (2.02%); industrial pizza (1.84%); industrial desserts (0.87%)^(4)^. Thus, the database consisted of all foods in these categories that are known to contribute 53.79% of mean adult energy intake in the UK^(4)^.

**Search strategy to identify UPF**

Every food item in these top 15 groups contributing to UPF intake were identified from an online search of supermarkets. The supermarket websites of the ‘‘big four’’ supermarkets that dominate the market share in the UK were reviewed, comprising Tesco (26.9% market share), Sainsbury’s (14.9%), Asda (13.9%) and Morrisons (10.1%) (data in the 12 weeks ending 14/06/2020 when the supermarket sample was selected)^(26)^.

A search term for each UPF group was devised (**Table 1**). As online supermarkets organise products into hierarchical ontologies, the search terms were chosen at the highest level in order to maximally widen the search (e.g. “bread” would capture white loaf, tortilla, wraps, ciabatta etc). Where food groups were broad descriptors (e.g. confectionary), numerous search terms were selected (e.g. sweets, chocolate) to maximise identification of individual food items^(25)^.

The search terms were used in the websites of the four supermarkets to retrieve all food products in the UK food supply in that group. The supermarket search was conducted in Google Chrome used in ‘incognito mode’ to minimise the influence of previous browsing history on retrieval of food brands from the search results^(27)^. Searches were conducted over a six-month period (June 2020 – November 2020) and was performed by a team of three research dietitians.

### *Data extraction for emulsifier content*

All retrieved food products from each supermarket search were transferred into the database ready for de-duplication, eligibility screening and data extraction.

Identical food products were identified, and duplicates discounted from the analysis (e.g. four identical brands of tomato ketchup in identical portion size, one from each of the supermarkets). Differing portion sizes of the same food products were not excluded on the basis of duplication (given the individual products remained representative of different products available in the food supply). After discounting duplicates, each food product was assessed for eligibility based on inclusion criteria (**Table 1**).

In the final count of eligible food products, the ingredients labels were extracted from supermarket database and manually reviewed for the presence of food-additive emulsifiers, and the details extracted. The list of 66 food-additives classified as emulsifiers was based on both JECFA and Codex Alimentarius classifications, and have been published elsewhere^(19)^, a list of which are provided for reference in **Supplementary Table 1**, including the International Numbering System code (“E number”) and full name of the food additive emulsifier.

Thus, the final database contained the following extracted data: food item brand name; food group (e.g. industrialised breads); search term under which it was identified, eligibility (yes/no); ingredients list for eligible food items; emulsifier details (presence/absence, which emulsifiers, total number of emulsifiers).

De-duplication, eligibility screening and data extraction was performed by the same team of three research dietitians. In order to ensure consistency of data extraction, the lead dietitian (PhD in emulsifiers and the gut) trained the team on the process and regularly reviewed data extraction in the early stages until the other team members developed independence. Following full data extraction, database checking was performed on 20 randomly selected food items from each of the 15 food groups (300 food items in total). Of the 300 random food items checked, there were no errors or disputes in de-duplication, eligibility screening or data extraction for emulsifiers.

### *Statistical analysis*

Frequency of emulsifiers are presented as median and interquartile range (IQR) due to non-normal distribution, and the number of emulsifiers in food products across UPF groups were compared using a Kruskal-Wallis test. Categorical data are presented as n (%) and were compared across UPF groups using Chi-square tests. Emulsifier co-occurrence was investigated using Spearman’s rank correlation and presented as a heatmap. For all statistical tests, a p-value of <0.05 was considered statistically significant.

## Results

### *Supermarket food product sample*

A total of 32,719 food products were returned in searches. Of these, 9,921 food products were excluded based on duplication and a further 9,952 excluded based on ineligibility (**Figure 1**). Therefore, the ingredients of 12,844 food products (both foods and beverages) in the UK supermarket food supply were manually reviewed for the presence of food-additive emulsifiers.

### *Occurrence of emulsifiers in UPFs*

Overall, food-additive emulsifiers were present in 6,642 (51.7%) of the 12,844 foods reviewed (**Table 2**).

The five most common emulsifiers across all products were lecithin (E322) (23.4% of all UPF), mono- and diglycerides of fatty acids (E471) (14.5%), diphosphates (E450) (11.6%), xanthan gum (8.2%) and pectin (E440) (8.0%) (**Table 2**).

From the 66 food-additive emulsifiers approved for use in the EU, 51 were identified in the products reviewed. Due to foods containing multiple emulsifiers (**Table 2**), there were 14,300 occurrences of emulsifiers across 6,642 emulsifier-containing foods. Patterns of occurrence of individual emulsifiers are shown in **Table 3**.

Of the emulsifiers with most research on their impact on the gastrointestinal tract, carrageenan was present in 565 foods (4.4%), E466 in 179 foods (1.4%) and polysorbate-80 in 8 foods (0.06%).

### *Occurrence of emulsifiers by food group in UPFs*

Overall, 51.7% of the foods contained food-additive emulsifiers, however there was wide variation and statistically significant differences between UPF groups (p < 0.001). For example, emulsifiers were contained in 95.0% of ‘‘Pastries, buns and cakes’’; 81.9% of ‘‘Milk-based drinks”, 81.0% in ‘‘Industrial desserts’’ and in 77.5% of “Confectionary”. In contrast, they were contained in only 11.6% of ‘‘Packaged salty snacks” (**Table 2**).

The type of emulsifier was also highly variable across food groups, for example, carrageenan (E407) was the most common emulsifier in ‘‘Milk-based drinks’’ (63.1% of products) and was only present in 4.9% of ‘‘Sausage and other reconstituted meat products’’ (**Table 2**). Carboxymethylcellulose (E466) was present in 40.0% of ‘‘Milk-based drinks’’ and only 3.0% of ‘‘Soft drinks, fruit drinks and fruit juices’’ (**Table 2**). Polysorbate-80 was only identified in 8 foods (**Table 3**) from varying food groups.

### *Frequency of emulsifiers in each UPF*

Across all 6,642 foods that contained an emulsifier, there were a median of 2 emulsifiers (IQR 2) per product (**Table 2**). Fifty-one percent of emulsifier-containing foods contained multiple emulsifiers, but this pattern of co-occurrence varied between food groups (p < 0.001) (**Table 2**). For example, emulsifier-containing ‘‘Pastries, buns and cakes’’ had a median of 4 emulsifiers (IQR 3) per product, and ‘‘industrialised packaged breads’’, ‘‘biscuits’’ and ‘‘milk-based drinks’’ had a median of 2 emulsifiers per product. Of all emulsifier-containing foods, 11.3% contained at least two emulsifiers, 6.1% three, 4.1% four and 4.8% five or more emulsifiers (**Figure 2**).

### *Co-occurrence of emulsifiers in UPFs*

There were a number of statistically significant positive correlations (Spearman’s rank) between emulsifier co-occurrence in foods indicating common use of the two emulsifiers together in a food product (**Figure 3**). There were strong positive correlations between the presence of polysorbate-60 (E435) and sorbitan monostearate (E491) (rho=0.70, p<0.001) and between carob bean gum (E410) and guar gum (E412) (rho=0.54, p<0.001). There were moderate positive correlations between the presence of ammonium salts of phosphatidic acid (E442) and polyglycerol esters of interesterified ricinoleic acid (E476) (rho=0.46, p<0.001); sodium lactylates (E481) and tragacanth gum (E413) (rho=0.32, p<0.001); microcrystalline cellulose and powdered cellulose (E460) and carboxymethyl cellulose (E466) (rho=0.47, p<0.001); and between E471 and E475 (rho=0.31, p<0.001).

There were a small number of statistically significant negative correlations between emulsifier co-occurrence, although in general these were weakly correlated (**Figure 3**). For example, lecithin (E322) and xanthan gum (E415) (rho=-0.22, p<0.001); indicating the presence of one was associated with less use of the other.

## Discussion

This paper demonstrates the ubiquity of food-additive emulsifiers across foods contributing to UPF intake in the UK food supply. Over half of foods in these UPF categories contained emulsifiers, being most commonly found in industrial desserts, milk-based drinks and confectionary, pastries and cakes. The most common emulsifiers were lecithin, mono- and diglycerides of fatty acids, diphosphates, xanthan gum and pectin, however, variations in emulsifier type occurred across food groups. Half of all emulsifier-containing foods contained more than one emulsifier, with two foods containing 11 emulsifiers.

Comparing the findings of the present study with previous literature is challenging as minimal data are available on the occurrence of emulsifiers in foods at the brand-level. Studies either attempt to measure emulsifier intakes in population groups or they measure emulsifier content in the food supply (as in the current study).

In terms of measuring emulsifier intakes, Shah *et al.,* (2017) estimated population exposure to 7 common emulsifiers in the US (carboxymethycellulose (E466), polysorbate-80 (E433), lecithin (E322), mono- and diglycerides of fatty acids (E471), sodium lactylates (E481), sucrose esters of fatty acids (E473) and polyglycerol esters of interesterified ricinoleic acid (E476)^(29)^. They combined national dietary survey data, including the 2003-2010 National Health and Nutrition Examination Survey (NHANES) (2-day dietary intake), and assumed all food categories contained the US Maximum Permitted Levels of each emulsifier^(29)^. Lecithin (55 mg/kg bw/d) and mono- and diglycerides of fatty acids (80 mg/kg bw/d) had the highest mean exposure, whereas exposure to carboxymethycellulose (27 mg/kg bw/d) and polysorbate-80 (8 mg/kg bw/d) was relatively low. Whilst a very different methodological approach was used to estimate emulsifier exposure by Shah *et al.,* (2017) (individual dietary intake data) compared to the present study (occurrence in the food supply), it mirrors the findings reported here that, lecithin and mono- and diglycerides of fatty acids are more common than polysorbate-80 or carboxymethycellulose.

Other studies have measured emulsifiers in the food supply, similar to the current study. The United States Department of Agriculture Global Branded Food Products Database (241,688 food products) has been used to investigate patterns of food-additive content across baked goods, reporting emulsifiers being present in 91% of cookies, 94% of crackers, 95% of bread and rolls and 100% of pastry and doughnuts^(30)^. Lecithin accounted for ~44–45% of the total counts of emulsifiers listed in cookies and crackers, whereas gums such as xanthan, guar, and agar account for ~18% of the listed emulsifiers in pastries. Whilst that study only measured food-additives in one food group, it concurs with the findings presented here that emulsifiers are ubiquitous in baked goods. A database study in France using The Open Food Facts Database (126,566 food products), found that emulsifiers/thickeners were one of the most frequently used food additives, compared with antioxidants, dyes, preservatives and sweeteners^(31)^, with the most commonly present emulsifiers being lecithin (2^nd^ most common food additive), xanthan gum (6^th^), diphosphates (8^th^), pectins (9^th^) and mono- and diglycerides of fatty acids (10^th^), which were identical to the five most common emulsifiers in the present study. In addition, carrageenan was common in the French food supply (13^th^ most common food additive, in 4.2% of foods), and is similar to that in the current study (4.4% of foods)^(31)^. Other studies have examined the presence of emulsifiers in food supply, although not at the level of individual emulsifiers as in the current study. A review of 24,229 foods in Australia reported emulsifiers to be the 7^th^ most common ingredient being present in 15.6% of all foods^(32)^, whilst a study of 9,856 foods in Brazil reported emulsifiers to be the 6^th^ most common food additive being present in 19.4% of all foods^(33)^. Both of these values are lower than that reported here (51.7%), although in the current study only foods contributing to UPF intake were selected and therefore our value is inevitably higher.

The emulsifiers implicated in gastrointestinal inflammation, carrageenan (4.4% of foods), carboxymethycellulose (1.4%) and polysorbate-80 (0.06%), were not overall very common in the UK food supply. However, carrageenan and carboxymethylcelluose, which form a viscous solution in the aqueous phase creating a stable water-fat emulsion for a creamy mouthfeel^(34,35)^, were therefore contained in 63.1% (carrageenan) and 40.0% (carboxymethylcellulose) of all milk based-drinks. Thus, whilst these additives are not ubiquitous in the food supply, people consuming high levels of these specific food groups may be disproportionately exposed. A recent human study reported that carboxymethylcelluose reduced microbial diversity^(36)^, meanwhile several prospective cohort studies report that higher intakes of UPF are associated with an increased risk of IBD^(37,38,14)^. Interestingly, different UPF categories, including soft drinks, refined sweetened foods, salty snacks, and processed meat were each independently associated with increased risk of IBD, suggesting that perhaps certain processing or food-additives commonly found in these groups (such as the aforementioned emulsifiers) could theoretically be contributing to the development of IBD^(14)^.

Polysorbate-80 was rarely identified in food products in the present study. This could explain why the European Food Safety Authority (EFSA) estimates of mean intakes of polysorbates across Europe (0.6 – 16.9 mg/kg bw/d)^(39)^ are lower than that of carrageenan (22.0 – 88.9 mg/kg bw/d)^(40)^ and the modified celluloses (20 – 67 mg/kg bw/d)^(41)^.

This is the first study to demonstrate that UPFs in the UK commonly contain multiple emulsifiers. Yet, whether emulsifiers have synergistic effects on gastrointestinal inflammation is not known, as previous murine and ex vivo studies tested individual emulsifiers in isolation^(21,22,42)^. Whilst half of all emulsifier-containing foods contained multiple emulsifiers, there were few strong patterns of co-occurrence. Co-occurrence of food additives is common across UPF. A third of foods in the United States Department of Agriculture Global Branded Food Products Database contain at least 3 additives^(43)^. The French-based Open Food Facts Database found that 11.6% of food products contained at least two additives, 7.8% three, 5.3% four and 11.3% five or more food additives^(31)^. This is similar to the present study, which found that 11.3% of foods contained at least two emulsifiers, 6.1% three, 4.1% four and 4.8% five or more emulsifiers. Of course, foods are often eaten together during a single meal, resulting in higher numbers, quantities and combinations of emulsifiers potentially being consumed within a single eating occasion. The NutriNet Santé cohort was used to identify six clusters of food additive consumption based upon foods commonly consumed together (e.g. additives found in breakfast cereals, pastries and dairy desserts)^(44)^, emphasising that foods containing emulsifiers and other food additives are often eaten together and therefore exposure will depend not only on food supply but also on food preference, choice and meal pattern.

Fifteen food-additives classified as emulsifiers were not present in the sample of UPF. This could be due to a number of reasons. Firstly, it could be that these emulsifiers are used in foods groups not in the top 15 most highly consumed UPF categories, although this would still result in low exposure. Secondly, it could be that food industry is reducing its use of certain emulsifiers. For example, soybean hemicellulose (E426) which was not present in any food products in this study, may be used less frequently since soy allergy has become better understood and reported^(45)^. Another example is alginic acid (E400), a gel forming additive, that has been identified as a choking hazard in jelly confectionary owing to its semi-rigid consistency, and so its use in foods has been limited by the EU^(1)^.

This study was the first to explore the occurrence of all food-additive emulsifiers in a large sample of foods in the UK food supply. The EFSA attempts to estimate population exposure to food additives in the EU, but current estimates use the rudimentary approach of assuming that all food categories that are permitted to contain emulsifiers will contain emulsifiers^(19)^. Whereas brand-level data with actual emulsifier occurrence would increase the external validity of population exposure estimates when combined with national dietary surveys, providing the surveys also record food intake at the brand-level.

Emulsifier occurrence in this sample of 12,844 foods was considerable. This is significant because more than half of all foods in UK households are ultra-processed^5^ and these foods contribute to more than half of the UK energy intake^(4)^. If the deleterious effects of emulsifiers observed in animal models are confirmed in humans, and the present study has highlighted the ubiquity of emulsifiers in the food supply, then food formulations would require significant changes. This study is the first to confirm the wide-spread occurrence of food-additive emulsifiers in the UK food supply and therefore the likely challenge of restricting dietary intakes of emulsifiers as part of ongoing therapeutic dietary interventions, for example, for metabolic syndrome^(23)^ or IBD^(20)^. Most concerningly, availability of UPFs in middle-income countries are growing rapidly. Sales of UPFs in South-East Asia and the East are expected to match those of high-income countries by 2035^(46)^.

### *Strengths, limitations and future research*

This is the first study to report the occurrence of emulsifiers in the UK food supply. It surveyed a large number of foods (12,844) contributing to UPF intake, larger than some previous studies in other countries^(30,33)^, although smaller than others that used pre-established databases^(31,32)^. Occurrence of total and individual emulsifiers was reported here whereas some previous studies report only the occurrence of ‘total emulsifiers’^(30,32,33)^, which is important as there is escalating evidence of differential impacts of individual emulsifiers on gut health^(42, 47)^.

A number of limitations of this study must be considered, and these mostly pertain to the UPF category sampling method.

Firstly, this study only sampled the top 15 food groups of the 16 possible UPF categories that contribute to energy intake in the UK^(4)^. Therefore, it is likely other foods not sampled here represent further sources of emulsifiers in the UK food supply. The only UPF category not sampled, the 16^th^ and final of the UPF groups contributing to energy intake, was “Miscellaneous”^(4)^. This was because it would be challenging to search, identify and correctly categorise brand level food items to a “miscellaneous” category using supermarket websites. In addition, potentially eligible UPFs in other categories were not searched. However, the top 15 UPF categories sampled collectively contribute to 53.8% of mean adult energy intake, and the remaining unaccounted miscellaneous group contribute only an additional 3.08% to total energy intake^(4)^.

Secondly, food items from only 4 supermarkets were analysed, and this does not represent the full range of food product availability. However, these ‘‘big four’’ supermarkets collectively dominate the UK food supply, owning the total market share of 65.8%^(26)^. Similarly, foods from restaurants or takeaways were not included in the analysis, and foods from these outlets may contain emulsifiers.

Thirdly, there is a risk the search terms did not retrieve all relevant foods and so some foods in these 15 UPF categories may have been missed. To minimise this, search terms were intentionally broad and inclusive in order to maximise the retrieval of results. Where it was perceived that a single search term would not be a catch-all term for a food group, multiple search terms for that food category were created *a priori* (**Table 1**).

Alternative data collection methodologies were considered that did not pose these three limitations in sampling approach. One option was to obtain the sample from market research data on the best-selling UPF products, as adopted to estimate phosphorus-based additives in the Australian food supply^(48)^ or using supermarket loyalty card data of food purchasing habits, as used to estimate protein intake in older adults in the UK^(49)^. The benefit of obtaining commercial data on UPF sales is that food products with a high population exposure are better captured in the sample frame. However, data on brand-level food sales was prohibitively costly, and this approach would not achieve the aim of this study, which related to measuring emulsifier occurrence in all foods contributing to UPF intake in the UK food supply.

Data were collected in the second half of 2020, just after the UK left the European Union (31^st^ Jan 2020) but during the COVID-19 pandemic. Therefore, although the data collection period was entirely post-Brexit, its proximity to Brexit and to a global pandemic, may mean that food supply measured at that time may have subsequently changed. Importantly, we surveyed four major supermarkets online (not in store) to minimise any supermarket-specific or localised food supply issues.

Future research may consider the nutrient composition, availability, and cost of emulsifier-free and emulsifier-containing foods to understand potential nutritional and economic impacts of consuming UPF containing emulsifiers. In addition, the database can be used in future to identify other food additives in the UK food supply, and techniques that adopt algorithmic approaches to identifying these, rather than manual data extraction, could expedite this process.

## Conclusion

Findings from this study demonstrate the wide-spread occurrence of food-additive emulsifiers in the UK supermarket food supply. Emulsifiers are present in 51.7% of foods from UPF categories, the most common being lecithin, diphosphates and mono- and diglycerides of fatty acids. This is the first study to demonstrate that UPFs in the UK commonly contain multiple emulsifiers, with 26.4% containing two or more emulsifiers. The three emulsifiers of interest in gastrointestinal inflammation are present in relatively small numbers of UPFs; however, 63.1% and 40.0% of milk-based drinks contained carrageenan and carboxymethylcelluose, respectively.

**Table 1: Search terms and eligibility criteria for the 15 top consumed ultra-processed food categories in the UK.** Adapted using the FAO descriptors of ultra-processed foods and National Diet and Nutrition Survey descriptors.

| **Food category contributing to UPF intake in UK** | | **Search term(s)** | **Included** | **Excluded** |
| --- | --- | --- | --- | --- |
| 1 | Industrialised packaged breads | Bread | All industrialised packaged breads including sliced bread, soda bread, bagels, pitta, baguettes, bread rolls, chapatis, naan, garlic bread, English muffins, wheat tortillas/wraps, brioche and part-baked packaged bread. Gluten-free versions of these breads were included. | Pizza, croutons, waffles, bread sauce mix, bread sticks, bread flour, bread and butter pudding, breadcrumbs, crisp bread crackers, bruschetta, poppadums, scones, hot cross buns, tea cakes, fruit loaf, malt loaf. |
| 2 | Packaged pre-prepared meals | Ready meal | All fresh or frozen packaged pre-prepared meals. Savoury meat pies were included (but reconstituted meat products on their own belong in group 4). | Packaged pre-prepared dishes that are side dishes (such as creamed carrots). Infant weaning ready meals. Nutritionally complete meal replacement drinks. Industrial pizza (group 14). |
| 3 | Breakfast cereals | Breakfast cereal | All breakfast cereals including porridge pots, breakfast slices (e.g. Kellogg’s® pop tarts), breakfast cereal bars (e.g. Kellogg’s Special K® bars and Alpen® breakfast bars) and breakfast biscuits (e.g. Belvita® breakfast biscuits). | Breakfast drinks (e.g. Weetabix® on-the-go drinks). Infant breakfast cereal (e.g. Aptamil® breakfast cereal 7 months+). Plain oats. |
| 4 | Sausage and other reconstituted meat products | Meat | Chilled, cured, frozen or canned reconstituted meat. Examples include sausage meat, chicken Kievs, burgers, meat balls, nuggets, pate, black/white pudding, pepperoni, pastrami, corned beef, luncheon meat, meat paste, faggots, and haggis. | Meats that have not been reconstituted (e.g. pre-prepared sliced chicken breast or minced meat) or meats that have been cured but not reconstituted (e.g. gammon or bacon). Meat alternatives. Fish and reconstituted fish. Reconstituted meats for pet foods. Reconstituted meat as part of a ready-meal (e.g. savoury meat pie, group 2). |
| 5 | Confectionary | Sweets  Chocolate | Boiled sweets, gums, pastilles, fudge, chews, mints, rock, liquorice, toffees, nougat. chocolate bars, filled bars, chocolate wafer bars, assortments, marshmallow-based products (e.g. Tunnock’s® teacakes), Florentines, chewing gum, diabetic chocolate, chocolate coated nuts/raisins. | Cereal bars or rice/corn cakes with chocolate (group 6). |
| 6 | Biscuits | Biscuit | All sweet and savoury biscuits, chocolate biscuits, Jaffa cakes, cereal bars, flapjacks, ice cream cornet/wafers, cream crackers, bread sticks, oat/rice cakes, macaroons. Gluten-free versions of these biscuits were included. | Cereal bars that are 100% dried fruit/nut (e.g. Nakd® bars). Infant weaning biscuits. |
| 7 | Pastries, buns and cakes | Pastry  Cake | All sweet pastry, pies and buns, pies. This includes Danish pastries, currant bun, doughnuts, American muffins, Eccles cakes, Bakewell tarts, jam tarts, scones (sweet and savoury), sponge cakes, fruit cakes, eclairs, fruit loaf, malt loaf, gateaux, pastry, mince pies, sponge fingers, scotch pancakes, croissants, custard tart, lemon meringue pie, egg custard, caramel shortcake, strudel. Ready-to-roll chilled/frozen pastry. | Chilled set puddings where the major component is not pastry/cake (e.g. jelly-set trifle, Tiramisu, cheesecake, banoffee pie). |
| 8 | Industrial chips (i.e. Oven chips, French fries) | Chips | French fries, croquettes, potato smileys, potato waffles, hash brown. Products may be fresh/ frozen and oven/microwave preparations. | Mashed potato powder (e.g. Smash®). |
| 9 | Soft drinks, fruit drinks and fruit juices | Soft drink  Fruit juice | All fruit juice, squashes, cordial. | Blended fruit-based drinks that contain milk (group 10). Alcohol-free or low alcohol wines/beers/ciders. |
| 10 | Milk-based drinks | Milkshake  Smoothie | Milk-based drinks based on animal milks. Milkshakes or smoothies marketed as high protein drinks. | Plain milk (whether pasteurised, unpasteurised or ultra-heat treated). Infant formula (powder or ready-to-drink). Milk powders (e.g. milk/milkshake powders). Milkshakes or smoothies made from dairy-free, alternative milks. Meal replacement powders. |
| 11 | Margarine and other spreads | Margarine  Spread | Margarine, cooking spread, butter alternatives (e.g. vegan spread, dairy free spread). | Butter, spreadable cheese, other cooking fats and oils (lard, dripping), fish- and meat-based spread (e.g. pate, dips, sandwich filling). |
| 12 | Sauces, dressings and gravies | Sauce  Dressing  Gravy  Condiment  Vinaigrette | White sauces, cook-in-sauces (pesto, stir through sauces, pasta sauces), sauce mixes, tomato ketchup, Bovril/Marmite, chutney (including Branston pickles, Piccalilli), gravy, mayonnaise, salad cream and dressings (e.g. thousand island dressing, sweet chilli, Worcester, brown, HP, mint, sriracha), stock (dried, concentrate and liquid) | Pies, packaged ready meals, dried herbs and spices (including spice mixes and rubs), tomato puree, stuffing (dried or fresh), yeast, pickles (pickled vegetables such as onions, gherkins, kimchi, sauerkraut), dessert syrups and spreads (chocolate, strawberry, toffee). Foods in which sauce is not the main component of the product (e.g. beans in sauce, tinned fish in sauce, pasta 'n' sauce). |
| 13 | Packaged salty snacks | Crisps  Pretzels  Popcorn | Vegetable and legume-based crisps (such potato, corn, lentil, chickpea), popcorn (not sweet), twiglets, tortilla chips/snacks, pork scratchings, savory flavoured rice snacks, Bombay mix. | Sweet popcorn, tortillas wraps, naan bread, nuts and seeds, yogurt and chocolate coated rice snacks, biscuits. |
| 14 | Industrial pizza | Pizza | Frozen pizza, ready-made pizza, pizza bases, pizza baguettes/rolls. | Pizza sauces, pizza dough, dough balls, pizza flavoured crisps/nachos. |
| 15 | Industrial desserts | Dessert  Ice cream | Dairy based desserts, ice cream, mousses, chilled set puddings (such as jelly-set trifle, tiramisu, cheesecake, banoffee pie), custard, flavoured yoghurt, squirty cream, dessert sauces (maple syrup, strawberry, chocolate, caramel), ice cream roll/ice cream filled buns. | Confectionary (chocolate, pastilles), fresh/frozen fruit, cream, crème fraiche, evaporated milk, biscuits, cakes, sweet pies and pastries, plain yoghurt, savory puddings (Yorkshire/or meat), wafers and ice cream cones. |

*Food groups are the top 15 consumed UPF in the UK based on National Diet and Nutrition Survey data^4^. The search terms and eligibility are adapted from FAO descriptors of UPF^(2)^ and the National Diet and Nutrition Survey categories^(25)^.*

**Table 2: Occurrence of food-additive emulsifiers by ultra-processed food category in the UK food supply**

| **Ultra-processed food category** | **Products assessed, n** | **Products with at least one emulsifier, n (%)** | **Products with multiple emulsifiers, n (% of products assessed, % of products with emulsifiers)** | **Emulsifiers per emulsifier-containing products, median (IQR)** | **Maximum emulsifiers in a product, n** | **Top 5 most common emulsifiers,**  **E-number (n, %)** |
| --- | --- | --- | --- | --- | --- | --- |
| **Total UPF** | 12,844 | 6,642 (51.7%) | 3,397 (26.4%, 51.1%) | 2 (2) | 11 | E322 (3,010, 23.4%)  E471 (1,862, 14.5%)  E450 (1,496, 11.6%)  E415 (1,058, 8.2%)  E440 (1,025, 8.0%) |
| Industrialised packaged breads | 640 | 399 (62.3%) | 219 (34.2%, 54.9%) | 2 (1) | 4 | E472e (224, 35.0%)  E471 (187, 29.2%)  E481 (88, 13.8%)  E464 (62, 9.7%)  E415 (47, 7.3%) |
| Packaged pre-prepared meals | 1,410 | 364 (25.8%) | 101 (7.2%, 27.7%) | 1 (1) | 6 | E440 (102, 7.2%)  E450 (82, 5.8%)  E471 (57, 4.0%)  E415 (45, 3.2%)  E412 (41, 2.9%) |
| Breakfast cereals | 505 | 115 (22.8%) | 30 (5.9%, 26.1%) | 1 (1) | 5 | E322 (96, 19.0%)  E440 (17, 3.4%)  E450, (15, 3.0%)  E472e (11, 2.2%)  E471 (8, 1.6%) |
| Sausages and other reconstituted meat products | 1,015 | 474 (46.7%) | 172 (16.9%, 36.3%) | 1 (1) | 6 | E450 (356, 35.1%)  E452 (79, 7.8%)  E407 (50, 4.9%)  E412 (34, 3.3%)  E404 (31, 3.1%) |
| Confectionary | 2,083 | 1,615 (77.5%) | 558 (26.8%, 34.6%) | 1 (1) | 7 | E322 (1314, 63.1%)  E476 (276, 13.3%)  E442 (252, 12.1%)  E471 (204, 9.8%)  E414 (132, 6.3%) |
| Biscuits | 874 | 557 (63.7%) | 286 (32.7%, 51.3%) | 2 (1) | 6 | E322 (456, 52.2%)  E450 (160, 18.3%)  E476 (86, 9.8%)  E440 (62, 7.1%)  E471 (59, 6.8%) |
| Pastries, buns and cakes | 823 | 782 (95.0%) | 690 (83.8%, 88.2%) | 4 (3) | 10 | E471 (606, 73.6%)  E450 (568, 69.0%)  E322 (384, 46.7%)  E440 (265, 32.2%)  E415 (240, 29.2%) |
| Industrial chips | 142 | 41 (28.9%) | 10 (7.0%, 24.4%) | 1 (0) | 3 | E415 (23, 16.2%)  E450 (15, 10.6%)  E464 (8, 5.6%)  E461 (3, 2.1%)  E471 (3, 2.1%) |
| Soft drinks, fruit drinks and fruit juices | 644 | 104 (16.1%) | 30 (4.7%, 28.8%) | 1 (1) | 5 | E440 (40, 6.2%)  E445 (25, 3.9%)  E414 (23, 3.6%)  E415 (19, 3.0%)  E466 (19, 3.0%) |
| Milk-based drinks | 160 | 131 (81.9%) | 92 (57.5%, 70.2%) | 2 (2) | 5 | E407 (101, 63.1%)  E466 (64, 40.0%)  E460 (56, 35.0%)  E412 (18, 11.3%)  E415 (14, 8.8%) |
| Margarine and other spreads | 101 | 61 (60.4%) | 26 (25.7%, 42.6%) | 1 (1) | 4 | E322 (42, 41.6%)  E471 (42, 41.6%)  E404 (11, 10.9%)  E476 (5, 5.0%)  *No others* |
| Sauces, dressings and gravies | 1,833 | 543 (29.6%) | 119 (6.5%, 21.9%) | 1 (0) | 4 | E415 (380, 20.7%)  E412 (93, 5.1%)  E322 (99, 5.4%)  = E405 (25, 1.4%)  = E440 (25, 1.4%) |
| Packaged salty snacks | 767 | 89 (11.6%) | 10 (1.3%, 11.2%) | 1 (0) | 3 | E471 (40, 5.2%)  E322 (30, 3.9%)  E414 (9, 1.2%)  E415 (7, 0.9%)  E472E (3, 0.4%) |
| Industrial pizza | 332 | 140 (42.2%) | 66 (19.9%, 47.1%) | 1 (1) | 5 | E450 (53, 16.0%)  E452 (52, 15.7%)  E415 (25, 7.5%)  E464 (22, 6.6%)  E412 (18, 5.4%) |
| Industrial desserts | 1,515 | 1,227 (81.0%) | 988 (65.2%, 80.5%) | 1 (2) | 11 | E471 (596, 39.3%)  E412 (549, 36.2%)  E322 (542, 35.8%)  E410 (534, 35.2%)  E440 (405, 26.7%) |
| P-value |  | <0.001 | <0.001 | <0.001 |  |  |

Ultra-processed food groups are defined as detailed by Rauber *et al.* (2018) according to the Food and Agriculture Organisation NOVA definitions^(4)^. Emulsifiers are identified by E-number (the Codex Alimentarius International Numbering System number, prefixed by the letter ‘‘E’’)^(28)^. Number of emulsifiers per food product (median, IQR) was assessed for significance between UPF groups using a Kruskal-Wallis test. Proportions of food products containing emulsifiers between UPF groups was assessed using Chi-square tests for independence.

**Table 3: Occurrence of individual emulsifiers across ultra-processed food categories in the UK food supply**

| Emulsifier E-number | Frequency of emulsifier occurrence across ultra-processed food categories, n | | | | | | | | | | | | | | | |
| --- | --- | --- | --- | --- | --- | --- | --- | --- | --- | --- | --- | --- | --- | --- | --- | --- |
|  | 1 Breads | 2 Prepared meals | 3 Breakfast cereal | 4 Sausage, meat products | 5 Confectionary | 6 Biscuits | 7 Pastries, buns, cakes | 8 Chips | 9 Soft drinks, fruit drinks, juices | 10 Milk drinks | 11 Margarine, spreads | 12 Sauces, dressings, gravy | 13 Salty snacks | 14 Pizza | 15 Desserts | Total occurrence, n (%) |
| E322(i-iii) | 18 | 9 | 96 | 18 | 1,314 | 456 | 384 | 0 | 0 | 0 | 42 | 99 | 30 | 2 | 542 | 3010 (21.0%) |
| E339(i-iii) | 1 | 28 | 0 | 5 | 3 | 10 | 18 | 0 | 1 | 7 | 0 | 1 | 1 | 2 | 32 | 109 (0.8%) |
| E400 | 0 | 0 | 0 | 0 | 0 | 0 | 0 | 0 | 0 | 0 | 0 | 0 | 0 | 0 | 1 | 1 (0.0%) |
| E401 | 1 | 2 | 0 | 12 | 7 | 3 | 89 | 0 | 0 | 0 | 0 | 2 | 0 | 0 | 108 | 224 (1.6%) |
| E402 | 2 | 0 | 0 | 0 | 0 | 0 | 0 | 0 | 0 | 0 | 0 | 0 | 0 | 0 | 3 | 5 (0.0%) |
| E403 | 0 | 0 | 0 | 0 | 0 | 0 | 0 | 0 | 0 | 0 | 0 | 0 | 0 | 0 | 0 | 0 (0.0%) |
| E404 | 0 | 0 | 0 | 31 | 0 | 0 | 21 | 0 | 0 | 0 | 11 | 0 | 0 | 0 | 9 | 72 (0.5%) |
| E405 | 0 | 0 | 0 | 0 | 0 | 0 | 0 | 0 | 0 | 0 | 0 | 25 | 0 | 0 | 0 | 25 (0.2%) |
| E406 | 2 | 11 | 0 | 2 | 6 | 1 | 35 | 0 | 0 | 0 | 0 | 3 | 0 | 4 | 44 | 108 (0.8%) |
| E407 | 0 | 27 | 2 | 50 | 26 | 3 | 29 | 1 | 1 | 101 | 0 | 4 | 0 | 12 | 309 | 565 (4.0%) |
| E407a | 0 | 0 | 0 | 9 | 0 | 0 | 0 | 0 | 0 | 0 | 0 | 0 | 0 | 0 | 3 | 12 (0.1%) |
| E410 | 0 | 8 | 0 | 7 | 10 | 13 | 16 | 0 | 1 | 4 | 0 | 15 | 0 | 4 | 534 | 612 (4.3%) |
| E412 | 13 | 41 | 3 | 34 | 2 | 9 | 30 | 2 | 15 | 18 | 0 | 93 | 0 | 18 | 549 | 827 (5.8%) |
| E413 | 0 | 0 | 0 | 0 | 1 | 1 | 85 | 0 | 0 | 0 | 0 | 1 | 0 | 0 | 4 | 92 (0.6%) |
| E414 | 0 | 12 | 3 | 1 | 132 | 15 | 62 | 0 | 23 | 3 | 0 | 1 | 9 | 7 | 27 | 295 (2.1%) |
| E415 | 47 | 45 | 5 | 24 | 12 | 34 | 240 | 23 | 19 | 14 | 0 | 380 | 7 | 25 | 183 | 1058 (7.4%) |
| E416 | 0 | 0 | 0 | 0 | 0 | 0 | 0 | 0 | 0 | 0 | 0 | 0 | 0 | 0 | 0 | 0 (0.0%) |
| E423 | 0 | 0 | 0 | 0 | 0 | 0 | 0 | 0 | 0 | 0 | 0 | 0 | 0 | 0 | 0 | 0 (0.0%) |
| E425 | 0 | 0 | 0 | 0 | 0 | 3 | 0 | 0 | 0 | 0 | 0 | 0 | 0 | 0 | 2 | 5 (0.0%) |
| E426 | 0 | 0 | 0 | 0 | 0 | 0 | 0 | 0 | 0 | 0 | 0 | 0 | 0 | 0 | 0 | 0 (0.0%) |
| E427 | 0 | 0 | 0 | 0 | 0 | 0 | 0 | 0 | 0 | 0 | 0 | 0 | 0 | 0 | 0 | 0 (0.0%) |
| E431 | 0 | 0 | 0 | 0 | 0 | 0 | 0 | 0 | 0 | 0 | 0 | 0 | 0 | 0 | 0 | 0 (0.0%) |
| E432 | 0 | 0 | 0 | 0 | 0 | 0 | 0 | 0 | 0 | 0 | 0 | 0 | 0 | 0 | 0 | 0 (0.0%) |
| E433 | 0 | 1 | 0 | 3 | 0 | 0 | 2 | 0 | 0 | 0 | 0 | 0 | 0 | 0 | 2 | 8 (0.1%) |
| E434 | 0 | 0 | 0 | 0 | 0 | 1 | 0 | 0 | 0 | 0 | 0 | 0 | 0 | 0 | 0 | 1 (0.0%) |
| E435 | 0 | 0 | 0 | 0 | 1 | 0 | 34 | 0 | 0 | 0 | 0 | 0 | 0 | 0 | 1 | 36 (0.3%) |
| E436 | 0 | 0 | 0 | 0 | 0 | 0 | 0 | 0 | 0 | 0 | 0 | 0 | 0 | 0 | 0 | 0 (0.0%) |
| E440 | 0 | 102 | 17 | 13 | 92 | 62 | 265 | 0 | 40 | 4 | 0 | 25 | 0 | 0 | 405 | 1025 (7.2%) |
| E442 | 0 | 0 | 0 | 0 | 252 | 28 | 12 | 0 | 0 | 0 | 0 | 0 | 1 | 0 | 53 | 346 (2.4%) |
| E445 | 0 | 0 | 0 | 1 | 0 | 1 | 0 | 0 | 25 | 0 | 0 | 0 | 0 | 0 | 0 | 27 (0.2%) |
| E450 (i-ix) | 35 | 82 | 15 | 356 | 21 | 160 | 568 | 15 | 0 | 0 | 0 | 5 | 8 | 53 | 178 | 1496 (10.5%) |
| E452 (i-vi) | 1 | 26 | 0 | 79 | 3 | 2 | 2 | 0 | 3 | 0 | 0 | 8 | 0 | 52 | 26 | 202 (1.4%) |
| E460 (i-ii) | 2 | 5 | 0 | 3 | 4 | 5 | 0 | 0 | 0 | 56 | 0 | 0 | 0 | 2 | 0 | 77 (0.5%) |
| E461 | 3 | 19 | 3 | 4 | 0 | 0 | 5 | 3 | 0 | 0 | 0 | 1 | 2 | 17 | 0 | 57 (0.4%) |
| E462 | 0 | 0 | 0 | 0 | 0 | 0 | 1 | 0 | 0 | 0 | 0 | 0 | 0 | 0 | 0 | 1 (0.0%) |
| E463 | 7 | 0 | 0 | 0 | 0 | 0 | 0 | 0 | 0 | 0 | 0 | 0 | 0 | 0 | 2 | 9 (0.1%) |
| E464 | 62 | 18 | 0 | 4 | 0 | 1 | 21 | 8 | 0 | 0 | 0 | 1 | 0 | 22 | 1 | 138 (1.0%) |
| E465 | 0 | 0 | 0 | 0 | 0 | 0 | 0 | 0 | 0 | 0 | 0 | 0 | 0 | 0 | 0 | 0 (0.0%) |
| E466 | 20 | 5 | 0 | 1 | 16 | 1 | 24 | 0 | 19 | 64 | 0 | 2 | 0 | 0 | 27 | 179 (1.3%) |
| E467 | 0 | 0 | 0 | 1 | 0 | 0 | 0 | 0 | 0 | 0 | 0 | 0 | 0 | 0 | 0 | 1 (0.0%) |
| E468 | 0 | 0 | 0 | 0 | 0 | 0 | 1 | 0 | 0 | 0 | 0 | 0 | 0 | 0 | 0 | 1 (0.0%) |
| E469 | 0 | 0 | 0 | 0 | 0 | 1 | 0 | 0 | 0 | 0 | 0 | 0 | 0 | 0 | 0 | 1 (0.0%) |
| E470 (i-iii) | 0 | 0 | 0 | 4 | 16 | 0 | 10 | 0 | 0 | 0 | 0 | 0 | 0 | 0 | 0 | 30 (0.2%) |
| E471 | 187 | 57 | 8 | 25 | 204 | 59 | 606 | 3 | 0 | 12 | 42 | 6 | 40 | 17 | 596 | 1862 (13.0%) |
| E472a | 0 | 0 | 0 | 0 | 0 | 3 | 4 | 0 | 0 | 0 | 0 | 0 | 0 | 0 | 3 | 10 (0.1%) |
| E472b | 0 | 0 | 1 | 0 | 3 | 2 | 35 | 0 | 0 | 0 | 0 | 0 | 0 | 1 | 95 | 137 (1.0%) |
| E472c | 0 | 0 | 0 | 0 | 0 | 1 | 8 | 0 | 0 | 1 | 0 | 0 | 0 | 0 | 6 | 16 (0.1%) |
| E472d | 0 | 1 | 0 | 0 | 0 | 0 | 33 | 0 | 0 | 0 | 0 | 1 | 0 | 0 | 4 | 39 (0.3%) |
| E472e | 224 | 15 | 11 | 6 | 1 | 25 | 3 | 0 | 0 | 0 | 0 | 0 | 3 | 4 | 7 | 299 (2.1%) |
| E472f | 0 | 0 | 0 | 0 | 0 | 0 | 9 | 0 | 0 | 0 | 0 | 0 | 0 | 2 | 0 | 11 (0.1%) |
| E473 | 0 | 8 | 0 | 0 | 17 | 8 | 17 | 0 | 0 | 0 | 0 | 0 | 0 | 0 | 1 | 51 (0.4%) |
| E474 | 0 | 0 | 0 | 0 | 0 | 0 | 0 | 0 | 0 | 0 | 0 | 0 | 0 | 0 | 0 | 0 (0.0%) |
| E475 | 2 | 6 | 7 | 0 | 4 | 14 | 231 | 0 | 0 | 0 | 0 | 0 | 0 | 3 | 66 | 333 (2.3%) |
| E476 | 2 | 0 | 0 | 0 | 276 | 86 | 32 | 0 | 0 | 0 | 5 | 0 | 1 | 0 | 82 | 484 (3.4%) |
| E477 | 0 | 1 | 0 | 0 | 0 | 0 | 37 | 0 | 0 | 0 | 0 | 0 | 0 | 0 | 32 | 70 (0.5%) |
| E478 | 0 | 0 | 0 | 0 | 0 | 0 | 0 | 0 | 0 | 0 | 0 | 0 | 0 | 0 | 0 | 0 (0.0%) |
| E479 | 2 | 0 | 0 | 0 | 0 | 0 | 0 | 0 | 0 | 0 | 0 | 0 | 0 | 0 | 0 | 2 (0.0%) |
| E481 (i-ii) | 88 | 1 | 0 | 0 | 0 | 7 | 133 | 0 | 0 | 0 | 0 | 0 | 0 | 0 | 7 | 236 (1.7%) |
| E482 (i-ii) | 1 | 0 | 0 | 0 | 0 | 0 | 0 | 0 | 1 | 0 | 0 | 0 | 0 | 0 | 0 | 2 (0.0%) |
| E491 | 0 | 0 | 0 | 0 | 0 | 0 | 22 | 0 | 0 | 0 | 0 | 0 | 1 | 2 | 0 | 25 (0.2%) |
| E492 | 0 | 0 | 1 | 0 | 29 | 8 | 26 | 0 | 0 | 0 | 0 | 0 | 0 | 0 | 2 | 66 (0.5%) |
| E493 | 0 | 0 | 0 | 0 | 0 | 0 | 0 | 0 | 0 | 0 | 0 | 0 | 0 | 0 | 0 | 0 (0.0%) |
| E494 | 0 | 0 | 0 | 0 | 0 | 0 | 0 | 0 | 0 | 0 | 0 | 0 | 0 | 0 | 0 | 0 (0.0%) |
| E495 | 0 | 0 | 0 | 0 | 0 | 0 | 0 | 0 | 0 | 0 | 0 | 0 | 0 | 0 | 0 | 0 (0.0%) |
| E541 | 0 | 0 | 0 | 0 | 0 | 0 | 0 | 0 | 0 | 0 | 0 | 0 | 0 | 0 | 0 | 0 (0.0%) |
| E999(i-ii) | 0 | 0 | 0 | 0 | 0 | 0 | 0 | 0 | 2 | 0 | 0 | 0 | 0 | 0 | 0 | 2 (0.0%) |
|  |  |  |  |  |  |  |  |  |  |  |  |  |  |  |  | **TOTAL 14300** |

Emulsifiers are identified by their E-number (the Codex Alimentarius International Numbering System number, prefixed by the letter ‘‘E’’)^(28)^ and the number of food products containing that emulsifier are reported for each of the top 15 UPF categories contributing to energy intake in the UK^(4)^.

**Figure legends**

**Figure 1: Flow diagram of food product inclusion**

**Figure 2: Number of individual emulsifiers per food product**

**Figure 3: Heatmap displaying co-occurrence of individual food-additive emulsifiers in UPF products in the UK food supply.** Values are Spearman’s rho correlation coefficients and cell shading indicates the magnitude of correlation co-occurrence between emulsifiers. The degree of green shading indicates positive correlations, red shading denotes negative correlations.

## References

1. European Commission (2008). Regulation No 1333/2008/EC of the European Parliament and of the Council of 16 December 2008 on Food Additives.
2. Monteiro CA, Cannon G, Lawrence M, *et al.* (2019). Ultra-processed foods, diet quality, and health using the NOVA classification system. Food and Agriculture Organization of the United Nations, Rome.
3. Martini D, Godos J, Bonaccio M, *et al*. (2021). Ultra-processed foods and nutritional dietary profile: a meta-analysis of nationally representative samples. *Nutrients*. 13(10):3390.
4. Rauber F, da Costa Louzada ML, *et al.* (2018). Ultra-Processed Food Consumption and Chronic Non-Communicable Diseases-Related Dietary Nutrient Profile in the UK (2008-2014). *Nutrients*. 10(5): 587.
5. Monteiro CA, Moubarac JC, Levy RB, *et al.* (2018). Household availability of ultra-processed foods and obesity in nineteen European countries. *Public Health Nutr.* *21*(1): 18–26.
6. Baraldi LG, Steele EM, Canella DS, *et al.* (2018). Consumption of ultra-processed foods and associated sociodemographic factors in the USA between 2007 and 2012: evidence from a nationally representative cross-sectional study. *BMJ Open*. 8(e020574).
7. Moubarac JC, Batal M, Louzada ML, *et al.* (2017). Consumption of ultra-processed foods predicts diet quality in Canada. *Appetite.* 108:512-520.
8. Machado PP, Steele EM, Levy RB, *et al.* (2019). Ultra-processed foods and recommended intake levels of nutrients linked to noncommunicable diseases in Australia: evidence from a nationally representative cross-sectional study. *BMJ Open*. 9(8), e029544.
9. Luiten CM, Steenhuis IH, Eyles H, *et al.* (2016). Ultra-processed foods have the worst nutrient profile, yet they are the most available packaged products in a sample of New Zealand supermarkets. *Public Health Nutr*. 19: 530–538.
10. Davidou S, Christodoulou A, Fardet A, *et al.* (2020). The holistico-reductionist Siga classification according to degree of food processing: an evaluation of ultra-processed foods in French supermarkets. *Food Func.* 11: 2026–2039.
11. Moradi S, Entezari MH, Mohammadi H, *et al*. (2023) Ultra-processed food consumption and adult obesity risk: a systematic review and dose-response meta-analysis. *Crit Rev Food Sci Nutr*. 63(2): 249-260.
12. Wang M, Du X, Huang W, *et al.* (2022) Ultra-processed Foods Consumption Increases the Risk of Hypertension in Adults: A Systematic Review and Meta-analysis. *Am J Hypertens*. 35(10): 892-901.
13. Isaksen IM, Dankel SN. (2023) Ultra-processed food consumption and cancer risk: A systematic review and meta-analysis. Clin Nutr. 42(6): 919-928.
14. Narula N, Chang NH, Mohammad D, *et al*. (2023) Food Processing and Risk of Inflammatory Bowel Disease: A Systematic Review and Meta-Analysis. *Clin Gastroenterol Hepatol*. 2023 (online ahead of print). doi: 10.1016/j.cgh.2023.01.012.
15. Taneri PE, Wehrli F, Roa-Díaz ZM, *et al.* (2022) Association Between Ultra-Processed Food Intake and All-Cause Mortality: A Systematic Review and Meta-Analysis. *Am J Epidemiol*. 191(7): 1323-1335.
16. Daniela Neri EM-S, Monteiro CA, Levy RB (2019). Consumption of ultra-processed foods and its association with added sugar content in the diets of US children, NHANES 2009–2014. *Ped. Obes*. 14, e12563.
17. Weaver CM, Dwyer J, Fulgoni VL, *et al.* (2014). Processed foods: contributions to nutrition. *Am J Clin Nutr*. 99 (6): 1525–1542.
18. Srour B, Kordahi MC, Bonazzi E, *et al.* (2022). Ultra-processed foods and human health: from epidemiological evidence to mechanistic insights. *Lancet Gastroenterol Hepatol*. 7(12): 1128-1140.
19. Cox S, Sandall A, Smith L, *et al.* (2021). Food additive emulsifiers: a review of their role in foods, legislation and classifications, presence in food supply, dietary exposure, and safety assessment. *Nutr Rev*. 79(6):726-741.
20. Bancil AS, Sandall AM, Rossi M, *et al.* (2021) Food Additive Emulsifiers and Their Impact on Gut Microbiome, Permeability, and Inflammation: Mechanistic Insights in Inflammatory Bowel Disease. *J Crohns Colitis*. 15(6):1068-1079.
21. Chassaing B, Koren O, Goodrich JK, *et al.* (2015). Dietary emulsifiers impact the mouse gut microbiota promoting colitis and metabolic syndrome. *Nature*. 519(7541): 92–96.
22. Chassaing B, Van de Wiele T, De Bodt J, *et al.* (2017). Dietary emulsifiers directly alter human microbiota composition and gene expression ex vivo potentiating intestinal inflammation. *Gut*. 66 (8): 1414–1427.
23. De Siena M, Raoul P, Costantini L, *et al.* (2022). Food Emulsifiers and Metabolic Syndrome: The Role of the Gut Microbiota. *Foods.* *11*(15): 2205.
24. Halmos EP, Mack A, and Gibson PR (2019). Review article: emulsifiers in the food supply and implications for gastrointestinal disease*.* *Aliment Pharmacol Ther*. 49(1): 41–50.
25. National Diet and Nutrition Survey (2011). Diet and Nutrition Survey of Infants and Young Children. Appendix M. Main and Subsidiary Food Groups. Medical Research Council Human Nutrition Research.
26. Kantar (2020). Grocery Market Share UK 12 weeks ending. Website accessed 14.06.2020: <https://www.kantarworldpanel.com/grocery-market-share/great-britain>
27. Satvat K, Forshaw M, Hao F, *et al.* (2014). On the privacy of private browsing - A forensic approach. *J. Inf. Secur.* Appl. 19 (1): 88–100.
28. FAO & WHO (2017). CODEX Alimentarius: International Food Standards. Class names and the international numbering system for food additives. Report no. CAC/GL 36-1989.
29. Shah R, Kolanos R, DiNovi MJ, *et al.* (2017). Dietary exposures for the safety assessment of seven emulsifiers commonly added to foods in the United States and implications for safety. *Food Addit Contam Part A Chem Anal Control Expo Risk Assess*. 34 (6): 905–917.
30. Ahuja J, Li Y, Bahadur R, *et al.* (2020). Characterizing Ingredients in Baked Products Sold in the U.S. *Current Developments in Nutrition.* 4 (2): 733.
31. Chazelas E, Deschasaux M, Srour B, *et al.* (2020). Food additives: distribution and co-occurrence in 126,000 food products of the French market. Sci Rep. 10(1):3980.
32. Gaines A, Shahid M, Huang L, *et al.* (2021) Deconstructing the Supermarket: Systematic Ingredient Disaggregation and the Association between Ingredient Usage and Product Health Indicators for 24,229 Australian Foods and Beverages. *Nutrients*. 13(6): 1882.
33. Montera VDSP, Martins APB, Borges CA, *et al.* (2021) Distribution and patterns of use of food additives in foods and beverages available in Brazilian supermarkets. *Food Funct*; 12(17): 7699-7708.
34. Blakemore WR and Harpel AR (2010). Carrageenan. In: Imeson A (ed.). Food Stabiliser, thickeners and gelling agents. Blackwell Publishing Ltd, New Jersey, USA. 72–94.
35. Murray JCF (2009). Cellulosics. In: Phillips GO and Williams PA (eds.). Handbook of hydrocolloids, 2nd edition. CRC Press, Boca, Raton. pp. 710–723.
36. Chassaing B, Compher C, Bonhomme B, *et al.* (2022) Randomized Controlled-Feeding Study of Dietary Emulsifier Carboxymethylcellulose Reveals Detrimental Impacts on the Gut Microbiota and Metabolome. Gastroenterology. 162(3):743-756.
37. Chen J, Wellens J, Kalla R, *et al.* (2022). Intake of ultra-processed foods is associated with an increased risk of Crohn's disease: a cross-sectional and prospective analysis of 187,154 participants in the UK Biobank*. J Crohns Colitis*. doi: 10.1093/ecco-jcc/jjac167.
38. Lo CH, Khandpur N, Rossato SL, *et al.* (2022). Ultra-processed foods and risk of Crohn's disease and ulcerative colitis: a prospective cohort study. *Clin Gastroenterol Hepatol*. 20(6):e1323-e1337.
39. European Food Safety Authority (2015). Scientific Opinion on the re-evaluation of polyoxyethylene sorbitan monolaurate (E 432), polyoxyethylene sorbitan monooleate (E 433), polyoxyethylene sorbitan monopalmitate (E 434), polyoxyethylene sorbitan monostearate (E 435) and polyoxyethylene sorbitan tristearate (E 436) as food additives. *EFSA J*. 13 (7): 1 -74.
40. European Food Safety Authority (2017). Re-evaluation of celluloses E 460(i), E 460(ii), E 461, E 462, E 463, E 464, E 465, E 466, E 468 and E 469 as food additives. *EFSA J*. 16(1):1 - 104.
41. European Food Safety Authority (2018). Re-evaluation of carrageenan (E407) and processed Eucheuma seaweed (E407a) as food additives. *EFSA J*. 16 (4): 5238.
42. Sandall AM, Cox SR, Lindsay JO, *et al.* (2020). Emulsifiers impact colonic length in mice and emulsifier restriction is feasible in people with Crohn's disease. *Nutrients*. 12(9):2827.
43. Tseng M, Grigsby CJ, Austin A, *et al.* (2022). Sensory-related industrial additives in the US packaged food supply. *Front Nutr.* 8:762814.
44. Chazelas E, Druesne-Pecollo N, Esseddik Y, *et al.* (2021) Exposure to food additive mixtures in 106,000 French adults from the NutriNet-Santé cohort. *Sci Rep*. 11(1): 19680.
45. Jędrusek-Golińska A, Piasecka-Kwiatkowska D, Zielińska P, *et al.* (2019). Soy preparations are potentially dangerous factors in the course of a food allergy. *Foods*. 8(12): 655.
46. The Global Panel on Agriculture and Food Systems for Nutrition (2016). Food systems and diets: Facing the challenges of the 21^st^ century. London, UK.
47. Naimi S, Viennois E, Gewirtz AT, *et al.* (2021) Direct impact of commonly used dietary emulsifiers on human gut microbiota. Microbiome. 9(1):66.
48. McCutcheon J, Campbell K, Ferguson M, *et al.* (2015). Prevalence of phosphorus-based additives in the Australian food supply: a challenge for dietary education? *J Ren Nutr*. 25(5):440-4.
49. Green MA, Watson AW, Brunstrom JM, *et al.* (2020). Comparing supermarket loyalty card data with traditional diet survey data for understanding how protein is purchased and consumed in older adults for the UK, 2014-16. *Nutr J*. 19(1): 83.
