# Supplementary material for: Emulsifiers in ultra-processed foods in the UK food supply
Source: Public Health Nutr. 2023 Sep 21;26(11):2256–70. doi: 10.1017/S1368980023002021 (PMC10641632; doi:10.1017/S1368980023002021)
Supplement: Supplementary file 1 [file S1368980023002021sup.zip › S1368980023002021sup002.docx]

**Emulsifiers in ultra-processed foods in the United Kingdom food supply**

**Alicia Sandall, Leanne Smith, Erika Svensen, Kevin Whelan**

**Supplementary Table 1 Food additive emulsifier numbers and names as presented in this manuscript.** These consist of the International Numbering System (INS) (“E number”) and the full name of all food additive emulsifiers as defined by the Joint Food and Agriculture Organization/World Health Organization Expert Committee on Food Additives (JECFA)

| **INS (E Number)** | **Food additive emulsifier** |
| --- | --- |
| E322(i-iii) | Lecithin |
| E339(i-iii) | Sodium phosphates |
| E400 | Alginic acid |
| E401 | Sodium alginate |
| E402 | Potassium alginate |
| E403 | Ammonium alginate |
| E404 | Calcium alginate |
| E405 | Propylene glycol alginate |
| E406 | Agar |
| E407 | Carrageenan |
| E407a | Processed euchema seaweed |
| E410 | Carob bean gum |
| E412 | Guar gum |
| E413 | Tragacanth gum |
| E414 | Gum arabic |
| E415 | Xanthan gum |
| E416 | Karaya gum |
| E423 | Octenyl succinic acid modified gum arabic |
| E425 | Konjac flour |
| E426 | Soybean hemicellulose |
| E427 | Cassia gum |
| E431 | Polyoxyethylene (40) stearate |
| E432 | Polyoxyethylene (20) sorbitan monolaurate (polysorbate 20) |
| E433 | Polyoxyethylene (20) sorbitan monooleate (polysorbate 80) |
| E434 | Polyoxyethylene (20) sorbitan monopalmitate (polysorbate 40) |
| E435 | Polyoxyethylene (20) sorbitan monostearate (polysorbate 60) |
| E436 | Polyoxyethylene (20) sorbitan tristearate (polysorbate 65) |
| E440 | Pectins |
| E442 | Ammonium salts of phosphatidic acid |
| E445 | Glycerol esters of rosin |
| E450 (i-ix) | Diphosphates |
| E452 (i-vi) | Polyphosphates |
| E460 (i-ii) | Celluloses |
| E461 | Methyl cellulose |
| E462 | Ethyl cellulose |
| E463 | Hydroxypropyl cellulose |
| E464 | Hydroxypropyl methyl cellulose |
| E465 | Methyl ethyl cellulose |
| E466 | Sodium carboxymethyl cellulose |
| E467 | Ethyl hydroxyethyl cellulose |
| E468 | Cross-linked sodium carboxymethylcellulose |
| E469 | Sodium carboxymethyl cellulose enzymatically hydrolysed |
| E470 (i-iii) | Salts of fatty acids, with base of Ca, Mg, K, Na, Al, NH3 |
| E471 | Mono- and di glycerides of fatty acids |
| E472a | Acetic and fatty acid esters of glycerol |
| E472b | Lactic and fatty acid esters of glycerol |
| E472c | Citric and fatty acid esters of glycerol |
| E472d | Tartaric acid esters of mono- and di- glycerides of fatty acids |
| E472e | Diacetyltartaric and fatty acid esters of glycerol |
| E472f | Succinylated monoglycerides |
| E473 | Sucrose esters of fatty acids |
| E474 | Sucroglycerides |
| E475 | Polyglycerol esters of fatty acids |
| E476 | Polyglycerol esters of interesterified ricinoleic acid |
| E477 | Propylene glycol esters of fatty acids |
| E478 | Lactylated fatty acid esters of glycerol and propylene glycol |
| E479 | Thermally oxidised soya bean oil with mono- and diglycerides of fatty acids |
| E481 (i-ii) | Sodium lactylates |
| E482 (i-ii) | Calcium lactylates |
| E491 | Sorbitan monostearate |
| E492 | Sorbitan tristearate |
| E493 | Sorbitan monolaurate |
| E494 | Sorbitan monooleate |
| E495 | Sorbitan monopalmitate |
| E541 | Sodium aluminium phosphates |
| E999(i-ii) | Bone phosphate |
